# Mountain colonization precedes shifts away from bee pollination in Melastomataceae

**DOI:** 10.1111/nph.70273

**Published:** 2025-06-08

**Authors:** Constantin Kopper, Jürg Schönenberger, Agnes S. Dellinger

**Affiliations:** ^1^ Department of Botany and Biodiversity Research University of Vienna Rennweg 14 Vienna 1030 Austria

**Keywords:** abiotic environmental factors, elevational gradients, floral evolution, Melastomataceae, pollination syndromes, pollinator shifts

## Abstract

- Shifts among different groups of pollinators are central in the evolution of flowering plants, yet mechanisms underlying pollinator shifts remain unclear. Environment‐induced reduction in pollinator availability and hence efficiency may destabilize ancestral plant–pollinator interactions and trigger shifts to new, more efficient pollinators, but formal tests remain scarce.
- We used a series of phylogenetic comparative methods on 333 species of the pantropical family Melastomataceae to test whether elevation, latitude and climatic variables explain pollinator shifts and the distribution of floral traits governing pollen release.
- We find that shifts away from bee pollination to generalist insect and vertebrate pollination associate with occurrence in cooler and wetter mountain environments. Also, we show that mountain colonization repeatedly preceded and, hence, likely triggered shifts away from bee pollination. Furthermore, our results suggest that the evolution of floral traits (larger petals and pore sizes) facilitating pollen transfer by bees may have been critical for the initial colonization of mountains by bee‐pollinated species.
- By identifying environments conducive to pollinator shifts, our results do not only provide a much‐needed hypothesis for mechanisms underlying the evolution of different pollination systems but also confirm their validity through empirical testing. Whether environment‐induced evolutionary pollinator shifts are the norm across angiosperms remains to be explored.

## Introduction

Mutualistic interactions, such as between plants and their animal pollinators, are increasingly considered important for the evolution and maintenance of biodiversity (Schemske *et al*., 2009). Such interactions are unevenly distributed across the globe, with more diverse interaction systems commonly found at lower latitudes (Sinnott‐Armstrong *et al*., 2021). Considering pollination, there are, for instance, more functional groups of pollinators in the tropics than in temperate zones (Endress, 1996; Ollerton, 2017). Similarly, the importance and availability of different pollinator groups change across elevational gradients (Cruden, 1972; Warren *et al*., 1988; Primack & Inouye, 1993; Dellinger *et al*., 2021). Globally, diverse and abundant insect pollinator assemblages at low elevations are narrowed to fly‐dominated pollinator assemblages at high elevations (Arroyo *et al*., 1982; Warren *et al*., 1988; Primack & Inouye, 1993; Lefebvre *et al*., 2018; Adedoja *et al*., 2020; McCabe & Cobb, 2021). In the Neotropics, these elevational turnovers in pollinator assemblages have been shown to lead to a dominance of vertebrate (particularly hummingbird) pollination in montane cloud forests (Dellinger *et al*., 2023). These latitudinal and elevational patterns indicate a critical role of the abiotic climatic environment in the evolution and distribution of plant–pollinator interactions, but large‐scale ecogeographic assessments of pollination strategies within plant lineages are currently scarce.

Differences in the abiotic environmental tolerances of different groups of pollinators (Ollerton, 2017; Lefebvre *et al*., 2018) may impact their pollination efficiency and have been proposed as main drivers of evolutionary pollinator shifts (Cruden, 1972; Arroyo *et al*., 1982; Krömer *et al*., 2006; Dellinger *et al*., 2021). For example, the flower visitation activity of ectothermic insect pollinators, such as most bees, depends on warm ambient temperatures and dry conditions (Cruden, 1972; Brito & Sazima, 2012; McCallum *et al*., 2013; Classen *et al*., 2015, 2020; Cozien *et al*., 2019). By contrast, not only many groups of endothermic vertebrate pollinators (e.g. birds and bats) but also flies and bumblebees may forage under cool and wet environmental conditions common in mountain ecosystems (Bruggemann, 1958; McCallum *et al*., 2013; Sun *et al*., 2014; Woodard, 2017; Oyen & Dillon, 2018; Lawson & Rands, 2019). Thus, while ectothermic insects may be highly efficient pollinators in warm and dry environments, their relative pollination efficiency may be reduced (i.e. through reduced visitation rates) compared with vertebrates, flies and bumblebees under cool and moist conditions (Cruden, 1972; Dellinger *et al*., 2021). In theory, such environment‐induced reductions in pollination efficiency could lead to the destabilization of formerly successful ancestral pollination systems and allow for shifts to new pollinators that are more efficient in a given environment (Thomson & Wilson, 2008). In the tropics, for example, we may expect consistent shifts between bee and vertebrate pollination in lineages that have colonized mountains, with bee‐pollinated species at lower and vertebrate‐pollinated species at higher elevations (cloud forests; Cruden, 1972, Dellinger *et al*., 2021).

**Fig. 1:**
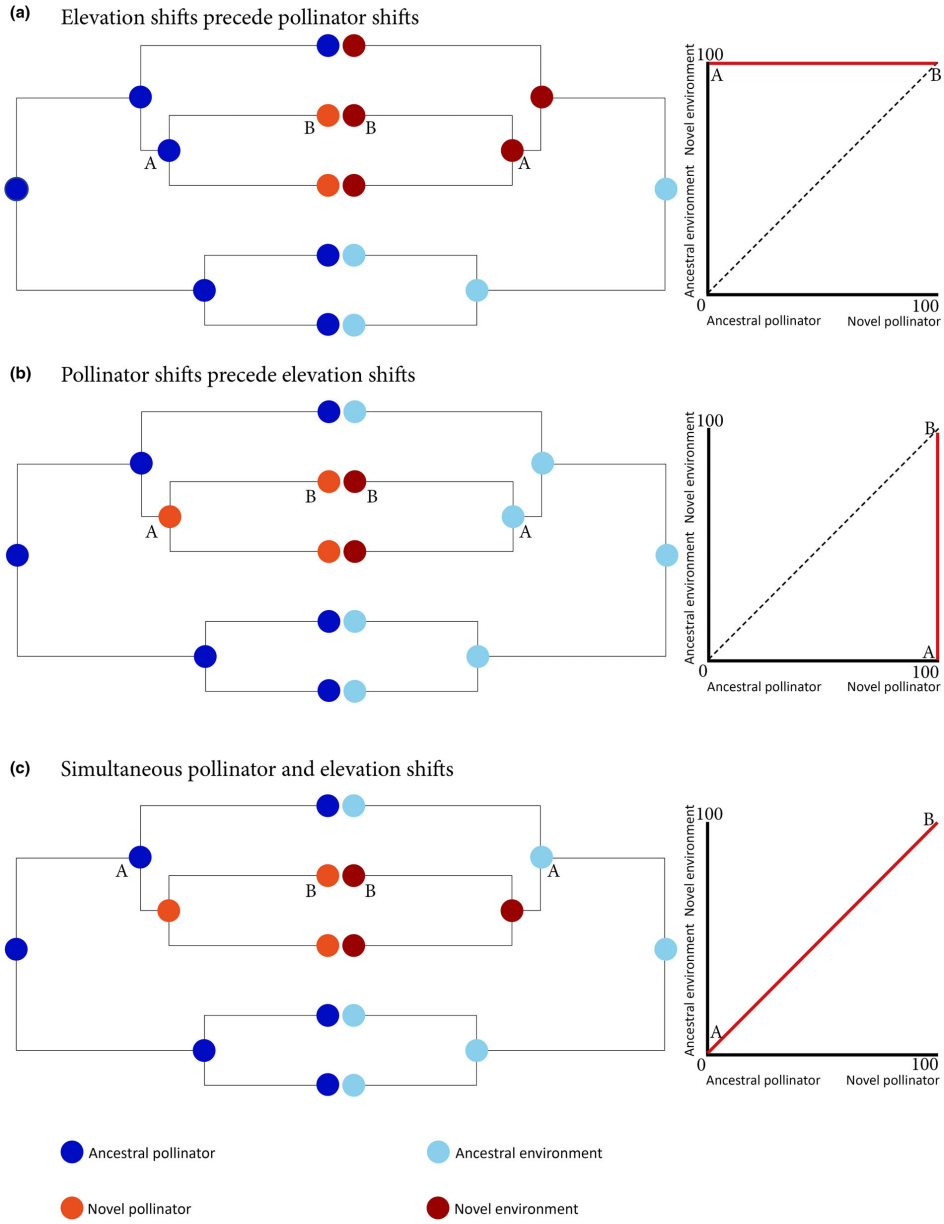
Schematic of the three different scenarios on the potentially interlinked nature of evolutionary pollinator shifts (left cladograms) and shifts into novel environments (right cladograms) and correlation plots for ancestral shift probability (right panels), where the *x*‐axis represents the ancestral node probability of having shifted pollinators and the *y*‐axis represents the ancestral node probability of having shifted environments. Dashed lines represent a 1 : 1 correlation, and red lines indicate expected values for ancestral nodes. (a) If environment shifts precede pollinator shifts, we expect shifts to novel environments to occur with a high probability at older nodes in a lineage's evolutionary history than pollinator shifts, and we would hence expect the observed values to lie above the 1 : 1 line in a correlation plot, with a high intercept indicating a high probability of having shifted to novel environments while still being pollinated by ancestral pollinators. (b) If pollinator shifts precede environmental shifts, we expect pollinator shifts to occur with a high probability at older nodes in the evolutionary history of a lineage than environment shifts, resulting in ancestral node values falling below the 1 : 1 correlation line and intercepts being negative, indicating a higher probability of having shifted pollinators while still being in the ancestral environment. (c) If pollinator and environment shifts occur more or less simultaneously and hence at similar nodes in the phylogeny, we expect empirical node values to fall around the 1 : 1 line in correlation plots, indicating joint shifts in environment and pollination system.

Although the ecogeographic patterning of pollination systems suggests reciprocity between the abiotic environment and evolutionary pollinator shifts (Arroyo *et al*., 1982; Lefebvre *et al*., 2018; McCabe & Cobb, 2021; Dellinger *et al*., 2023), their interlinked nature has rarely been explored macroevolutionarily (but see Hamilton & Wessinger, 2022, Dellinger *et al*., 2024). Combining information on pollination systems and the abiotic environment and mapping both on molecular phylogenies of plant clades allow us to trace the sequence of evolutionary events (i.e. pollinator shifts and environment shifts), and hence identify their relative timing and potential for reciprocal effects. Specifically, we may envision three scenarios for links between environment and pollinator shifts (Fig. 1). First, if environment‐induced reductions in the efficiency of ancestral pollinators are indeed a common theme in evolutionary pollinator shifts, we would expect shifts into novel environments (with unfavorable conditions for ancestral pollinators) to precede pollinator shifts (Fig. 1a). In such a scenario, plant species would first colonize novel environments (i.e. mountains), where reductions in the pollination efficiency of ancestral pollinators (i.e. bees) may induce shifts to novel, more efficient pollinators (i.e. birds). Support for such a scenario comes from the North American genus *Penstemon*, where bee‐ and hummingbird‐pollinated sister pairs occur in significantly different habitats compared with more distantly related bee‐pollinated species, suggesting that shifts into novel environments preceded pollinator shifts (Hamilton & Wessinger, 2022). Similarly, in the Melastomataceae tribe Merianieae, biogeographic range expansion into the Andes preceded shifts from bee to vertebrate pollination (Dellinger *et al*., 2024). Alternatively (Fig. 1b), shifts to novel pollinators may represent a critical precondition for range expansions into novel environments, and hence precede environmental shifts. Finally (Fig. 1c), pollinator shifts and environment shifts may occur more or less simultaneously (i.e. species are changing pollinators as they are expanding ranges) and should result in similar macroevolutionary patterns of correlated evolution. Empirical support for either of the latter hypotheses is currently lacking.

The two studies finding support for the scenario that environment shifts preceded pollinator shifts (Hamilton & Wessinger, 2022; Dellinger *et al*., 2024) also show that species with the ancestral pollination system were not only able to initially colonize novel environments but also persist there. Hence, the question arises why colonization of novel environments sometimes triggers pollinator shifts, and sometimes does not. Adaptive floral trait evolution might play a critical role in retaining ancestral pollinators even under unfavorable conditions (Galen, 1989; Bradshaw & Schemske, 2003; Smith & Kriebel, 2018; Dellinger *et al*., 2019a). Especially when ancestral pollinators occur in lower abundance in the newly colonized environment (i.e. fewer bee pollinators in mountains, Cruden, 1972, Dellinger *et al*., 2021), the evolution of traits maximizing pollen transfer even with the ancestral pollinators might be advantageous (Thomson *et al*., 2000). Such traits include investment in overall floral display (e.g. flower size), which is positively correlated with pollination rates across angiosperms (Dafni *et al*., 1997; Goodwillie *et al*., 2010). Traits regulating pollen dispensing (e.g. tempo and mode of anther dehiscence and pollen release), as well as the amount of pollen released at each pollinator visit, may also change with changing pollinator availability (Castellanos *et al*., 2006). Specifically, pollen may be dispensed in larger doses if the overall probability of pollinator visits is low or if pollinators ‘waste’ large proportions of the received pollen (e.g. bees actively collect pollen as fodder for their brood; Castellanos *et al*., 2006). Although highly relevant for understanding the mechanisms behind evolutionary pollinator shifts, these predictions stem from the study of few selected species only and have not incorporated the impact of different abiotic environmental contexts. It thus remains unclear whether trait modifications to maximize pollen transfer might generally represent an alternative to pollinator shifts when lineages colonize environments where their ancestral pollinators are rare. In such a scenario, we may expect selective pressure to maximize pollen transfer by increasing floral display and reducing pollen dosing traits (Dafni *et al*., 1997; Thomson *et al*., 2000; Castellanos *et al*., 2006; Goodwillie *et al*., 2010).

We here use the species‐rich, pantropical family Melastomataceae (5858 species in 173 genera and 23 tribes; Penneys *et al*., 2022) as a model to test whether environment shifts precede, follow or go hand‐in‐hand with pollinator shifts. The family is well suited for this assessment since it provides the required evolutionary replicates of environment and pollinator shifts. Specifically, our data encompass at least 11 independent environment shifts (across 128 out of 333 sampled species) from lowland rainforests to montane cloud forests, and at least 21 independent pollinator shifts (across 53 species from seven tribes) from ancestral buzz pollination by bees (most common, *c*. 96% of species) to nonbuzz pollination systems by vertebrates (i.e. birds, bats and rodents; Lumer, 1980, Dellinger *et al*., 2014, 2019b) or generalist insect pollinators (i.e. flies, wasps, beetles and butterflies; Kriebel & Zumbado, 2014, Brito *et al*., 2016). Melastomataceae are further characterized by poricidal anthers, whereby anthers only dehisce through a small apical pore (Endress, 1996). This stamen morphology effectively restricts the amount of pollen released at each pollinator visit (i.e. Larson & Barrett, 1999; Dellinger *et al*., 2019c), and across buzz‐pollinated lineages, pore size has been hypothesized as an important parameter controlling the amount of pollen released, with smaller pores strongly restricting pollen release (i.e. Kemp & Vallejo‐Marín, 2021; Boucher‐Bergstedt *et al*., 2025). In addition, Melastomataceae vary in the structure of their anther walls, which are either smooth or more or less corrugated, the latter potentially hindering pollen flow and further modulating pollen release (Dellinger *et al*., 2019c).

Comparing the elevational distribution and pollination systems (bee‐pollinated vs shifted) of 333 Melastomataceae species, we here first assess whether pollinator shifts do indeed associate with occurrence in cool and wet mountain environments across the family. Second, we reconstruct the evolutionary history of pollinator shifts and elevation shifts to test whether colonization of novel environments (mountains in our case) repeatedly preceded, and hence likely triggered, shifts away from bee pollination (Fig. 1a). If mountain colonization indeed likely preceded pollinator shifts, we expect to find shifts to mountains at older nodes in the Melastomataceae phylogeny than pollinator shifts so that closely related bee‐pollinated and shifted species both occur in mountains. If, instead, pollinator shifts represent a prerequisite for the colonization of mountain environments (Fig. 1b), we expect all bee‐pollinated species (regardless of whether they belong to tribes encompassing shifts or not) to occur at lower elevations and in warmer and drier, bee‐friendly habitats than shifted species. If, lastly, pollinator and environment shifts happened simultaneously (Fig. 1c), we expect pollinator shifts to mostly coincide with elevation shifts along the same nodes of the phylogeny and show correlated evolution. Given that we find strongest support for Scenario 1 (environment shifts preceding pollinator shifts), we further explore whether adaptive trait evolution potentially facilitating pollinator attraction and pollen transfer (larger pores and smooth anthers) may explain how some bee‐pollinated species could retain bee pollination in montane environments despite the reduced abundance of bee pollinators.

## Materials and Methods

We ran all statistical analyses in R (R Core Team, 2022); please find detailed methods in Supporting Information Notes S1.

### Species selection, pollination mode and phylogenetic hypothesis

Our analyses were based on a dataset of 333 species (*c*. 6% of Melastomataceae), for 206 of which floral visitors were empirically documented (Dellinger *et al*., 2022). For the remaining 127, we have predicted pollinators in a previous study using machine learning‐based classification methods (random forest analyses), where we trained initial models on 44 floral traits and 252 species with documented pollinators. Then, we used these trained and validated (high prediction accuracy) models to predict pollinators for the remaining species (Kopper *et al*., 2024). Of the 333 species used in this study, 53 species across seven tribes have shifted pollinators. These shifted species can be summarized into three pollination syndromes: ‘generalist’ (flies, wasps, beetles, butterflies and bees), ‘nectar‐foraging vertebrate’ (hummingbirds, flower piercers, lizards, rodents and bats) and ‘food‐body‐foraging vertebrate’ (passerine birds and parrots; Dellinger *et al*., 2022, Kopper *et al*., 2024). For most analyses, we merged these three pollination syndromes (all derived from the ancestral ‘buzz‐bee’ syndrome) into ‘shifted’ since we wanted to test whether there are general associations between shifts and environmental conditions. Also, this grouping was necessary to assure sufficient evolutionary replication.

We used the most recent dated molecular phylogeny for Melastomataceae (Reginato *et al*., 2022; 2454 tips, crown node age: 68.5–75.2 million years (Myr)) and pruned the maximum clade credibility (MCC) tree to the 333 species in our data matrix (see Notes S1). Throughout the manuscript, we use the term *tribe* to refer to the 23 taxonomic tribes recognized in Melastomataceae (Penneys *et al*., 2022), while we use the term *clade* for groups of closely related species *within* a tribe that all share a common ancestor, that is differentiating *pollinator‐shifted* and *nonshifted clades* within *tribes*. We define a shifted clade as a group of species whose last common ancestor and all of its descendants have shifted pollinators. Because we observe few or no reversals back to bee pollination, species descending from a lineage where a shift has occurred typically retain the new pollination system. Thus, a pollinator shift at the ancestral node of a *shifted clade* usually results in a clade composed entirely of species with shifted pollination systems. Note that *nonshifted clades* can exist within shifted *tribes* and be sister to *shifted clades*. *Nonshifted clades* are composed only of bee‐pollinated species and do not contain any descendants with a pollinator shift.

### GBIF occurrence data, elevation and climatic variables

We screened the initial plant species list (*n* = 336) using *Taxonstand* (u.taxonstand v.2.4; Zhang & Qian, 2023) to correct spelling mistakes and synonyms. Next, we submitted the list to GBIF (Global Biodiversity Information Facility) to search for occurrence data for each species (rgbif v.3.7.9; Chamberlain *et al*., 2024; 120 923 occurrence points). We excluded records lacking coordinates and submitted the resulting dataset to standard cleaning procedures (coordinatecleaner v.3.0.1; Zizka *et al*., 2019) to remove records located in country centroids, the sea, around GBIF headquarters, duplicates or records with equal longitude and latitude, leaving 88 932 records across 307 species. Since *Taxonstand* did not correct all misspelled names and synonyms, we manually corrected names, adding eight species (1158 occurrence points). Furthermore, 21 rare species had been excluded by our strict filtering settings; for these species, we searched occurrences with more relaxed settings, adding 4538 records and 18 species (three species were not found). We combined these datasets (resulting in 333 species and 94 628 occurrence points) and performed a final round of manual cleaning to ensure that all obtained records correspond to the known distribution ranges of the species. Specifically, for each species, we plotted range maps (maptools v.1.1.4; Bivand & Lewin‐Koh, 2023), compared these maps against the distributions documented in Plants of the World Online (https://powo.science.kew.org/; Fig. S1b) and removed records outside of the distribution range of the species (resulting in 90 498 records, 333 species and 22 tribes). Next, we subsampled occurrences so that each species was only represented by one occurrence in a 1 km grid. This left us with a final dataset of 71 122 occurrence records across 22 tribes and 333 species (Table S1), with a median number of 74 occurrence points per species (range: 1–3037). Across pollination strategies, bee‐pollinated species are represented by 63 911 records (90%) and shifted species are represented by 7211 records (10%; 4404 records of ‘generalist’, 2162 records of ‘nectar‐foraging vertebrate’ and 645 records of ‘food‐body‐foraging vertebrate’ syndrome).

To investigate the environmental context of pollinator shifts, we chose to focus on elevation, temperature and precipitation since all of these variables can significantly impact the flower visitation activity and abundance of bee pollinators and may hence be conducive to pollinator shifts (Cruden, 1972; Dellinger *et al*., 2023). Accordingly, we downloaded layers for mean annual temperature (bio1), mean annual precipitation (bio12, Notes S2) and elevation from chelsa v.2.1 (http://chelsa‐climate.org/) at 1‐km resolution (Karger *et al*., 2023). We extracted the respective value for each occurrence record and calculated the median value per species for analyses in a phylogenetic context. The final dataset is available in the online repository phaidra (https://phaidra.univie.ac.at/o:2096899). Note that elevation and mean annual temperature are strongly correlated (*r* > −0.8); we hence only included one at a time in our models (to avoid bias due to autocorrelation; Fig. S1a).

### Do shifts away from bee pollination associate with montane environments?

To assess whether high‐elevation occurrence is linked to nonbee pollination in Melastomataceae, we used boxplots to visualize the elevational distribution of bee‐pollinated and shifted species. We also plotted elevation across latitude for the different pollination systems using ggplot2 (v.3.5.1; Wickham, 2016). We applied phylogenetic ANOVAs (phytools, v.2.1.1; Revell, 2024) to test whether pollination systems differ in elevation. Next, to explore the impact of elevation, latitude and/or annual precipitation on pollination system, we fitted binary phylogenetic generalized linear mixed models (binaryPGLMM, *binaryPGLMM* and ape v.5.8; Paradis & Schliep, 2019). We built an initial binaryPGLMM, including interaction effects among elevation, latitude and precipitation. We used stepwise model selection to retain only significant parameters and interaction effects (elevation and latitude in our case) and plotted the expected value of the response variable to evaluate model fit (mu; Fig. S2a).

Since median values do not capture the range of environmental conditions that species with wider distribution ranges may encounter, we additionally performed a random sampling approach of the occurrence data to include environmental variables for single occurrence points per species. Specifically, 100 times, we randomly sampled a single occurrence point per species and reran binaryPGLMMs to evaluate the effect of elevation on pollination system. We calculated the percentage of runs where *P* values were smaller than 0.05 (indicating significant differences in the elevational distribution among pollination systems).

We used Whittaker biomes (Whittaker, 1975; Ricklefs, 2008; plotbiomes Ștefan & Levin, 2018) and the chi‐squared statistics to further explore whether bee‐pollinated and shifted species occur in different environments. We plotted standardized residuals of chi‐squared tests using correlation plots (corrplot v.0.92; Wei & Simko, 2024), with standardized residuals higher than ± 2 indicating a strong contribution of the respective biome to pollination system distribution.

### Did niche expansion into mountain habitats precede pollinator shifts?

**Fig. 2:**
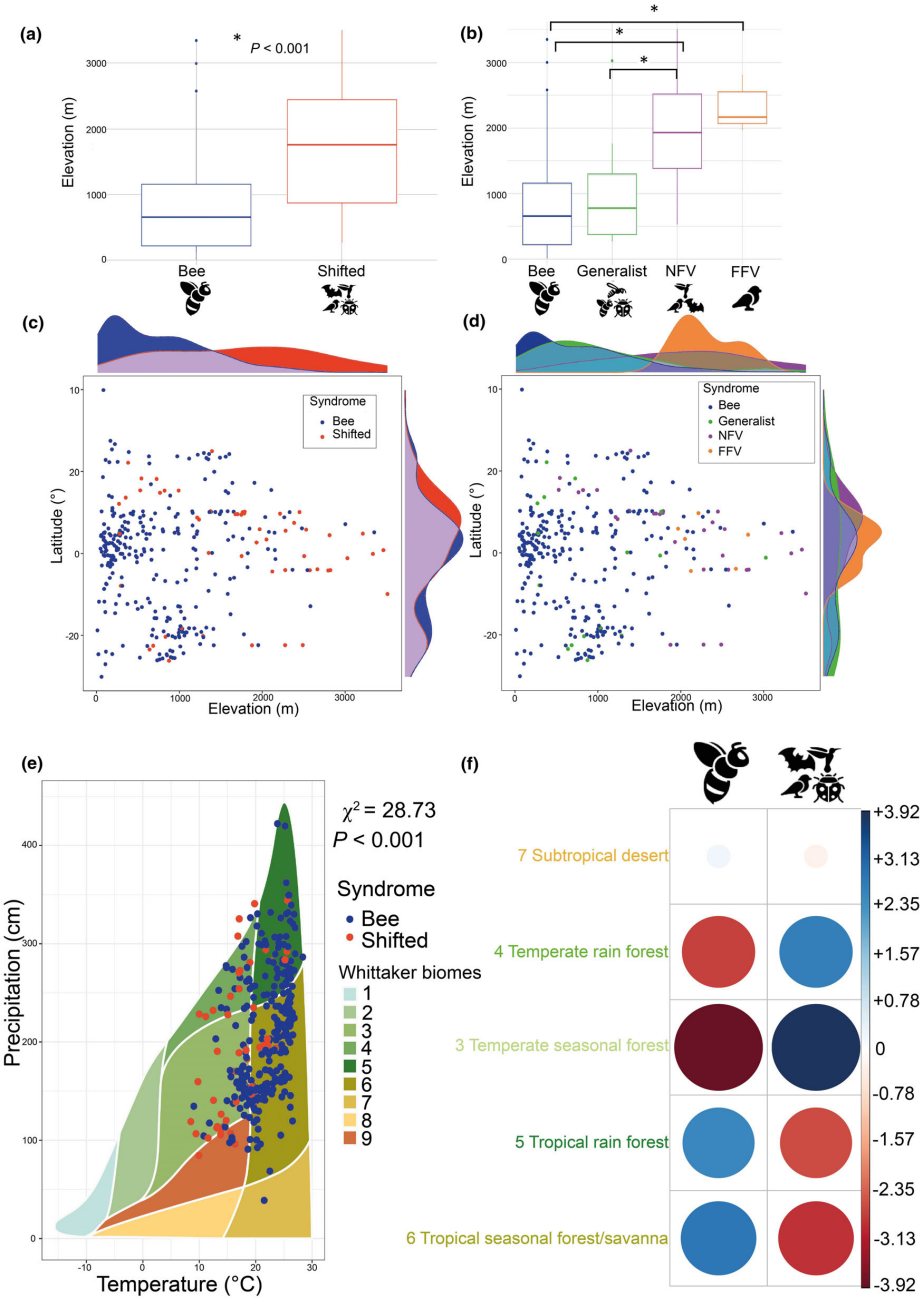
Melastomataceae species with shifted pollination systems occur at significantly higher elevations and in different biomes than bee‐pollinated species. (a) Bee‐pollinated species (blue) are more common below 1000 m while species that shifted pollinators (red) are more common above 1500 m. (b) Comparing all four pollination syndromes reveals that species that shifted to generalist pollination (green) are most common below 1000 m (like bee‐pollinated species (blue)), while all species that shifted to vertebrate pollination are significantly more common above 1500 m; boxes in (a) and (b) represent the interquartile ranges; bars inside the boxes represent the median values; whiskers represent the range and the dots represent outliers. Asterisk indicates significant differences. *P* values were obtained using phylogenetic ANOVA. (c, d) picture the elevation–latitude relation, showing that at higher latitudes, species that shifted away from bee pollination may occur at lower elevations. In (c), the comparison between bee‐pollinated and shifted species is shown (compare a), while (d) represents all four pollination syndromes (compare b). Density plots on top of (c, d) represent the interpolated elevational distribution; density plots on the right side of (c, d) represent the interpolated latitudinal distribution. (e) Whittaker biomes (Whittaker, 1975) are shown, with differently colored surfaces indicating different biomes (circumscribed by temperature (*x*‐axis) and precipitation (*y*‐axis)), and dots indicating species' means in temperature and precipitation. Bee‐pollinated species (blue dots) most commonly occur in tropical seasonal forests and savannas (Biome 6) and tropical rain forests (Biome 5), while shifted species (red dots) commonly occur in temperate rain forests (Biome 4) and temperate seasonal forests (Biome 3). (f) Plot of absolute standardized residuals of the chi‐squared test on the differences in biome occupation between bee‐pollinated and shifted species, with blue indicating a positive relationship, and red indicating a negative relationship. Size of the circles indicates the magnitude of the relationship; faded and small circles indicate a weak relationship. Numbers represent Whittaker biomes: 1, Tundra; 2, Boreal forest; 3, Temperate seasonal forest; 4, Temperate rain forest; 5, Tropical rain forest; 6, Tropical seasonal forest/savanna; 7, Subtropical desert; 8, Temperate grassland/desert; 9, Woodland/shrubland; Melastomataceae are absent from Biomes 1, 2 and 8.

To explore the three different scenarios of evolutionary pollinator and environment shifts (Fig. 1), we ran ancestral state reconstructions for both pollination system and elevation (which we determined as most important factor explaining the distribution of pollination systems). We chose to use the four different pollination syndromes (‘bee’, ‘nectar‐foraging vertebrate’, ‘food‐body foraging vertebrate’ and ‘generalist’) for the initial reconstruction of pollination systems since shifts into each syndrome require different modifications in floral morphology, and reconstructing them all together as ‘shifted’ might artificially inflate shift proportions at internal nodes. To make reconstructions comparable with elevation (a quantitative trait), we chose to categorize elevation into four broad categories: below 500, 500–1000, 1000–1500 m and *c*. 1500 m. This categorization is based on the observation that most pollinator shifts happen above 1000 m (Fig. 2). To ascertain that this categorization accurately mirrors elevation–pollination relationships as reported before, we repeated binaryPGLMMs using pollination system as binary response and categorized elevation as predictor.

For ancestral character estimation for pollination system, we ran three different MuSSE models (SYM, ARD and Constraint) using the function *make.musse* (diversitree v.0.10.0 FitzJohn, 2012) following Kopper *et al*. (2024). The constraints for transition rates were determined based on our knowledge of the family (Dellinger *et al*., 2022; see Notes S1). For elevation, we ran two different MuSSE models (SYM and ARD). We compared model fit using Akaike information criterion (AIC) scores (Table S2) and chose the model with the lowest score for either trait (SYM for pollination system and ARD for elevation). We also reconstructed elevation evolution as a continuous trait; results of these analyses can be found in the Supporting Information.

For visualizing evolutionary patterns on the phylogeny and calculating summary statistics, we binarized the pollination system (bee‐pollinated/shifted) and elevation (lowland/montane). Since elevation is a continuous variable and binarization is challenging, we employed three different binarization schemes: > 500 m – montane; > 1000 m – montane; and > 1500 m – montane. This approach allowed us to evaluate whether choosing different elevational thresholds for lowland/montane biased our results. We next extracted the reconstructed probability of having shifted pollinators and elevation, as well as node ages for all nodes descending from tips with pollinator shifts, until reaching 100% probability of being bee‐pollinated (ancestral condition). If only the tip showed 100% probability of having shifted, we used the tip values of this species. If sister species shared a lower node with 100% probability of having shifted, we used the value of the last shared node for this clade with 100% probability of having shifted. We plotted the extracted probabilities for each shifted clade (pollinator shift probability on the *x*‐axis and elevation shift probability on the *y*‐axis) for three different elevation binarizations. We further tested for correlated evolution between pollinator and elevation shifts using the function *fitCorrelationTest* (corhmm v.2.8; Beaulieu *et al*., 2013) using the four elevation categories and the four pollination syndromes.

To further explore the link between elevation and pollination system evolution, we tested three different hypotheses using Ornstein–Uhlenbeck models (OU models; l1ou, v.1.43; Khabbazian *et al*., 2016). First, we assigned separate elevationary optima for tribes containing species that have shifted pollinators and tribes that remained bee‐pollinated. If this model came out as the best fitting, it would further support the hypothesis that elevation shifts (whole tribes) precede pollinator shifts (within tribes; Fig. 1a). Second, we assigned separate optima for species that shifted pollinators and those that remained bee‐pollinated. If this model came out as the best fitting, it would support the hypothesis that pollinator shifts are a prerequisite for elevation shifts (Fig. 1b). Third, to explore the elevational relation of different pollination systems, we assigned two separate elevational optima, one for bee‐pollinated species and species that shifted to the ‘generalist’ syndrome and one separate optima for species that either shifted to the ‘nectar‐foraging vertebrate’ or to the ‘food‐body‐foraging vertebrate’ syndrome. We used *estimate_convergent_regimes* (l1ou, v.1.43; Khabbazian *et al*., 2016) to test for convergent elevational shifts across all models.

### Do bee‐pollinated species from shifted tribes occur in environments conducive to pollinator shifts?

To investigate whether bee‐pollinated relatives of shifted species are indeed more likely to occur in environments conducive to pollinator shifts (cool and wet mountain habitats, in our case) than bee‐pollinated species from nonshifted tribes, we analyzed the environmental associations between bee‐pollinated species only (*n* = 280). Using phylogenetic *t*‐tests and Whittaker biomes, we tested whether bee‐pollinated species from tribes containing shifts generally occur at higher elevations and cooler and wetter environments than bee‐pollinated species from tribes lacking shifts.

### Are bee‐pollinated species in mountains confined to more bee‐friendly environments?

To understand whether pollinator shifts may have allowed Melastomataceae to expand their niches into even harsher environments compared with species with the ancestral pollination system, we subset the full dataset to only contain species growing above 1000 m (the general threshold for pollinator shifts, *n* = 128). We built a binaryPGLMM to test whether mean annual temperature and precipitation can determine the probability of being bee‐pollinated or shifted among these montane species, with the expectation that shifted species may occur in even colder and wetter environments than their bee‐pollinated relatives (McCallum *et al*., 2013; Classen *et al*., 2015, 2020; Cozien *et al*., 2019). We used stepwise selection to select the parameters with significant effects on the pollination system and plotted the expected value of the response variable to assess model fit (Fig. S2b).

### Do montane bee‐pollinated species have larger flowers and traits facilitating pollen dispersal?

To explore whether montane bee‐pollinated species exhibit floral traits potentially increasing attractiveness to pollinators (larger petals) and facilitating pollen release with the scarce montane bee pollinators (smooth stamen walls and larger pores), we compared floral traits across bee‐pollinated species. Again, we split the dataset into lowland (< 1000 m) and montane (> 1000 m) species. We used phylogenetic linear mixed models (phylolm Ho & Ane, 2014) to test for the impact of elevation, mean annual precipitation, latitude and the interaction between latitude and elevation on petal length and pore size, and corresponding binaryPGLMMs for the structure of stamens (smooth or ruminate). We used trait values from Kopper *et al*. (2024). Pore size was determined using the total pore area (*A* = π × *a* × *b*), where ‘*a*’ represents half the pore height and ‘*b*’ represents half the pore width. We log‐transformed petal length and pore size to improve PGLMM fit and used stepwise model selection to identify significant variables.

## Results

### Shifts away from bee pollination are associated with cool montane environments

Following our expectations, species which have shifted away from ancestral bee pollination occurred at significantly higher elevations than bee‐pollinated species (Fig. 2a). This pattern was mostly consistent when lumping all shifted species into one category (Fig. 2a) and when retaining the four distinct pollination syndromes (Fig. 2b). Species from the ‘nectar‐foraging vertebrate’ and the ‘passerine’ syndromes generally occur above 1000 m and increase in abundance with increasing elevation (Fig. 2a,b). Species from the ‘generalist’ insect pollination syndrome can also occur at lower elevations (at *c*. 500 m), but with a peak in abundance at slightly higher elevations than bee‐pollinated species (Fig. 2d). Bee‐pollinated species were found along the whole elevational gradient, but their abundance decreased markedly with increasing elevation (Fig. 2c,d).

Using binaryPGLMMs, we found that the distribution of pollination systems was best explained by elevation and latitude (*P*_elevation_ = 1.2e−08, *P*_latitude_ = 0.02; Tables S3, S4; Figs 1a–d, S2a), but not precipitation. Species that shifted pollinators occurred at higher elevations around the equator than at higher latitudes (Fig. 2c,d). These results were consistent when resampling the dataset to include one randomly chosen occurrence per species, with 100% of resampling runs finding shifted species to have a significantly higher probability of occurring at high elevations than bee‐pollinated species.

Further exploring the association between pollination system and environment through Whittaker biomes, we found bee‐pollinated species in significantly different biomes than species which shifted pollinators (χ^2^ = 28.73, *P* = 8.87e−06; Fig. 2e,f). Bee‐pollinated Melastomataceae species grow more commonly in warm tropical rain forests and tropical seasonal forests, while shifted Melastomataceae species grow more commonly in cooler temperate rain forests and temperate seasonal forests. This differentiation in biome occupation among pollination systems is mostly driven by temperature rather than precipitation (Fig. 2e). As elevation and temperature are highly correlated (Fig. S1a), these results are in line with results from binaryPGLMMs identifying elevation as the most important explanatory variable in differentiating bee pollination from shifted pollination systems in Melastomataceae.

### Colonization of mountain habitats precedes pollinator shifts

**Fig. 3:**
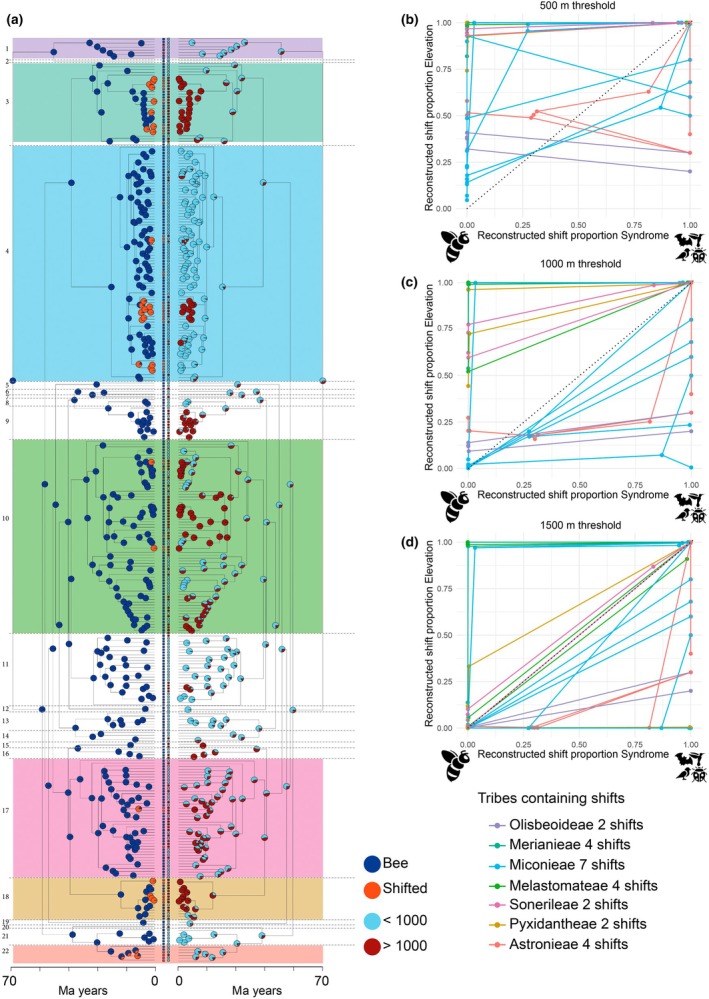
Ancestral character estimations of pollination systems (bee or shifted) and elevation showing that, generally, elevation shifts happened independently from pollinator shifts and that pollinator shifts usually happened after shifts to higher elevations. (a) The two dated phylogenetic trees show the reconstruction of pollination syndromes (left) using a MuSSE (SYM) model with nodes and tips colored as a binary trait (bee as blue and shifted as orange) and the reconstruction of elevation (right) based off a MuSSE (ARD) model, with nodes and tips colored in a binary manner with reconstructions < 1000 m summarized as ‘lowland’ and > 1000 m as ‘montane’. Pie charts at nodes represent probabilities of being bee‐pollinated (blue) or shifted (orange) and of being lowland (light blue) or montane (dark red); tip states for pollination system and elevation are also shown as pies. The seven tribes where pollinator shifts have occurred (1, 3, 4, 10, 17, 18 and 22) are highlighted with colors and correspond to colors used in Table 1 and (b–d). (b–d) The reconstructed shift proportions of pollination syndrome on the *x*‐axis and elevation on the *y*‐axis of tribes containing pollinator shifts (‘shifted tribes’) are shown; dots along the lines represent ancestral nodes descending from shifted tips (100% shift probability) to nodes with 0% shift probability (also compare Table 1); colors correspond to tribes as indicated in (a). All tribes are represented by at least two lines of the same color since all tribes contain at least two independent pollinator shifts. Following theoretical expectations from Fig. 1, a grouping of ancestral nodes along the *y*‐axis and lines above the 1 : 1 ratio supports elevation shifts preceding pollinator shifts, a grouping of ancestral nodes along the *x*‐axis and lines below the 1 : 1 ratio supports pollinator shifts preceding elevation shifts, while a grouping of nodes close to 0 and lines following the 1 : 1 ratio supports correlated evolution between elevation and pollinator shifts. (b) When all species occurring above 500 m were considered ‘montane’, 25 out of 25 ancestral nodes lie on the *y*‐axis and lines group above the 1 : 1 ratio. (c) When all species occurring above 1000 m were considered ‘montane’ (compare (a)), 19 out of 25 ancestral nodes lie on the *y*‐axis and six nodes lie close to 0. Thirteen out of 25 lines fall above the 1 : 1 ratio. (d) When choosing 1500 m as the cutoff for being ‘montane’, 10 ancestral nodes lie on the *y*‐axis, seven ancestral nodes lie close to 0 and 8 ancestral nodes lie along the *x*‐axis, and 10 out of 25 lines fall above the 1 : 1 ratio. Numbers on the very left in (a) indicate tribes: 1, Olisbeoideae; 2, Kibessieae; 3, Merianieae; 4, Miconieae; 5, Eriocnemeae; 6, Trioleneae; 7, Rupestreae; 8, Rhexieae; 9, Microlicieae; 10, Melastomateae; 11, Marcetieae; 12, Dinophoreae; 13, Dissochaeteae; 14, Cambessedesieae; 15, Stanmarkieae; 16, Cyphostyleae; 17, Sonerileae; 18, Pyxidantheae; 19, Bertolonieae; 20, Lithobieae; 21, Henrietteeae; 22, Astronieae; tribes containing species which shifted pollinators: 1, 3, 4, 10, 17, 18 and 22.

To investigate the interplay of evolutionary pollinator shifts and niche expansion into novel mountain environments, we reconstructed the evolutionary history of pollinator shifts and elevation. Across reconstruction methods (treating elevation as categorical or continuous trait), the root of Melastomataceae was reconstructed with a high probability as occurring in lowland conditions with bee pollination as the ancestral pollination system. Mountain colonization occurred repeatedly across Melastomataceae, both in tribes containing pollinator shifts and in purely bee‐pollinated tribes (Figs 3a, S3a). Bee‐pollinated lineages sister to species/lineages that have shifted pollinators were generally found in montane environments, supporting the hypothesis that shifts in elevation preceded pollinator shifts (Figs 1a, 3a, S3). In the tribes Olisbeoideae (two pollinator shifts) and Astronieae (three out of four pollinator shifts), pollinator shifts have occurred at relatively low elevations (around or below 500 m), while the five other tribes containing pollinator shifts (Melastomatoideae – four shifts, Merianieae – four shifts, Miconieae – seven shifts, Pyxidantheae – two shifts and Sonerileae –two shifts) showed shifts at or above 1000 m (Table 1).

**Table 1:** Reconstructed shift probability of pollination system and elevation at different nodes of tribes, which contain species that shifted pollinators.

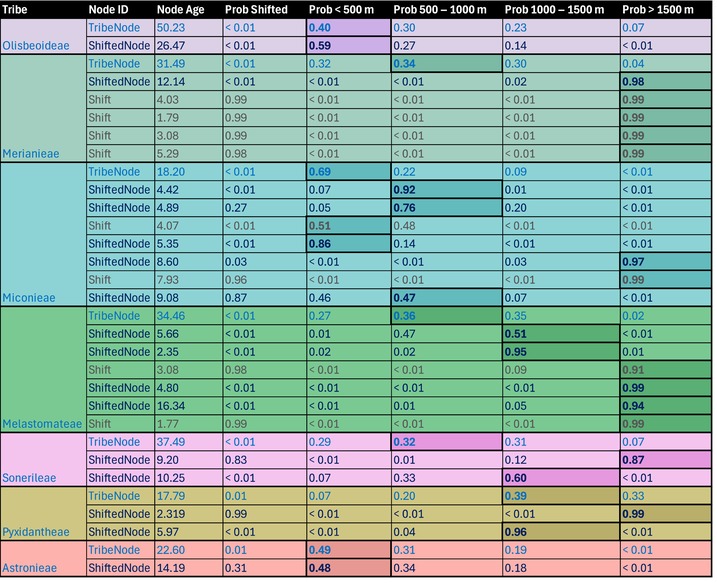

Tribe crown nodes (TribeNode) are usually located at lower elevations than nodes of shifted clades within a tribe (ShiftedNode). Nodes that show a high probability of having shifted pollinators (Shift) are usually located at higher elevations than ancestral nodes of a tribe (TribeNode) and nodes of shifted clades within a tribe (ShiftedNodes). Bold values with highlighted cell margins represent maximum values for elevation shifts. Colors correspond to tribes as used in Fig. 3. Prob Shifted gives the probability at a certain node of having shifted. Prob < 500, 500–1000, 1000–1500 and > 1500 m give the probabilities at a certain node of being in each respective elevation bin. Different colors in writing are further used to emphasize differences in nodes considered – light blue for tribe crown nodes (TribeNode), dark blue for nodes of clades encompassing pollinator shifts (ShiftedNode), and grey for nodes within clades containing pollinator shifts, with these nodes also having a high probability of having shifted (Shift) away from bee pollination.

Extracting reconstructed node values for elevation and pollination for all lineages, including pollinator shifts, we explored whether pollinator shifts likely occurred within montane environments (Fig. 1a), preceded mountain colonization (Fig. 1b) or went hand in hand with mountain colonization (Fig. 1c). We found strongest support for the first scenario (Fig. 1a), which shifts into mountain environments preceded shifts in pollinators. When the cutoff for ‘montane’ is set to 500 m, 25 of 25 lineages had intercepts above the 1 : 1 line, indicating a higher probability of having shifted to montane habitats while still being bee‐pollinated. Also, lines mostly group above the 1 : 1 line (Fig. 3b), indicating that shifts to mountains preceded pollinator shifts. When the cutoff is 1000 m, 19 of 25 lineages still had intercepts along the *y*‐axis, with 6 close to 0, suggesting a low probability of having shifted to mountains or pollinators, and 13 out of 25 lineages grouped above the 1 : 1 line (Fig. 3c). When the cutoff for being montane is 1500 m, the majority of lineages (10) had intercepts above the 1 : 1 line, 7 were close to 0, and 8 were following the *x*‐axis, indicating a higher probability of having shifted pollinators while still occurring below 1500 m. Additionally, 10 out of 25 lineages grouped above the 1 : 1 line (Fig. 3d). In support of the pattern that shifts to montane environments precede pollinator shifts, the last common ancestors of tribes (tribe crown nodes) where pollinator shifts have occurred (Table 1; TribeNode, likely bee‐pollinated) are commonly located below 1000 m, while within these tribes, the last common (likely bee‐pollinated) ancestors of shifted clades (Table 1; ShiftedNode) are commonly located at a higher elevation than tribe crown nodes. These findings again indicate that mountains were mostly colonized by species that were bee‐pollinated and pollinator shifts happened later in mountain environments.

We found no evidence for correlated evolution of elevation and pollination system (AIC dependent model = 1103.698, AIC independent model = 1011.816), again supporting the hypothesis that shifts in elevation and pollination system happened independently.

These results were confirmed when reconstructing elevation as a continuous trait, with 19 out of the 25 shifts across all tribes showing pollinator shifts in mountain environments and continued expansion into higher elevations in shifted clades (Fig. S3b). OU models further confirmed these findings, with Hypothesis 1 (separate elevational optima for entire tribes with shifted species, and hence also encompassing closely related bee‐pollinated species) resulting as best fit (Fig. S4a–c; Table S5).

### Bee‐pollinated species of shifted tribes occur in environments conducive to pollinator shifts

**Fig. 4:**
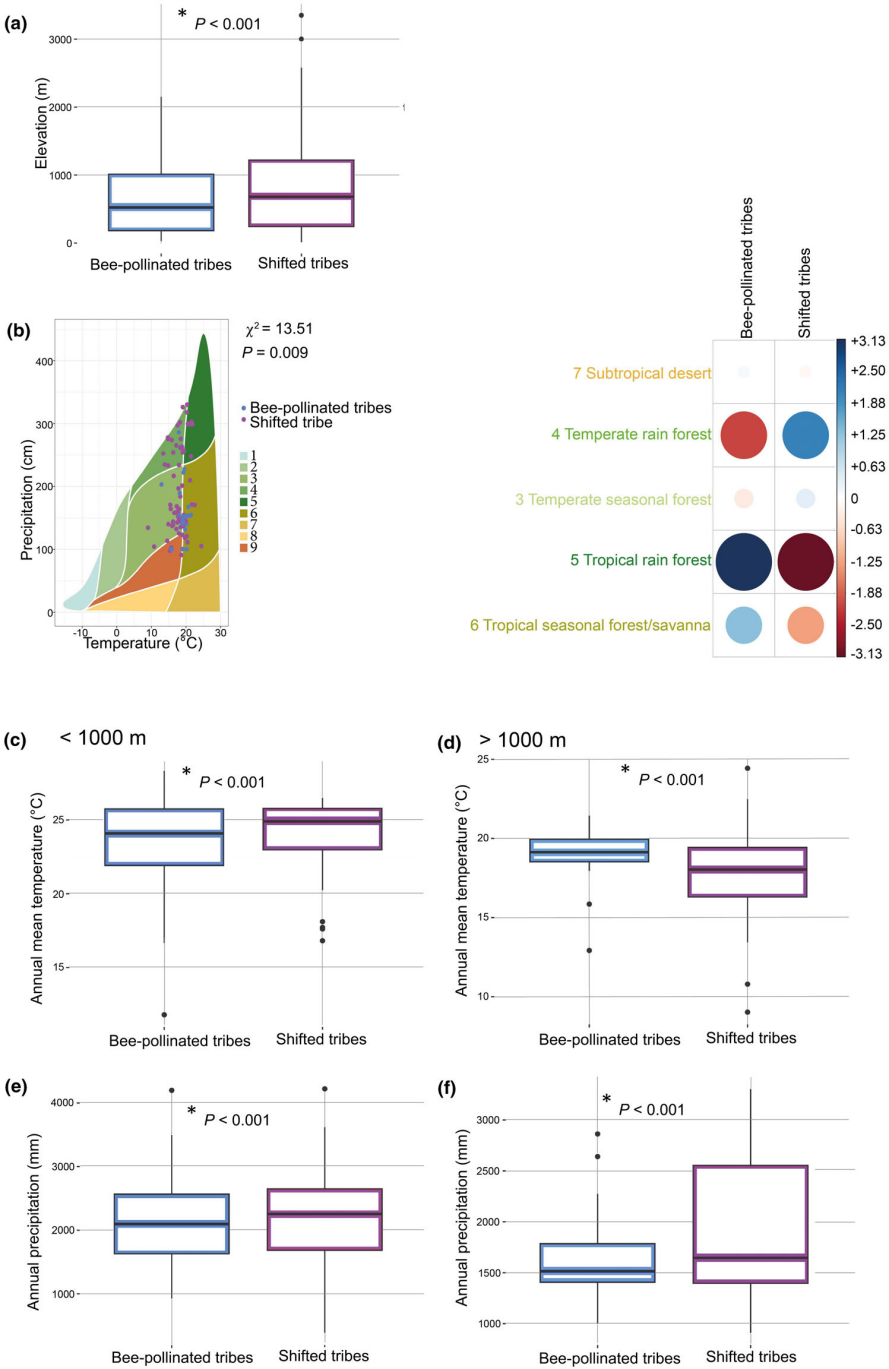
Bee‐pollinated species from tribes that contain pollinator shifts occur at higher elevations and under colder and wetter conditions than bee‐pollinated species from tribes without pollinator shifts. (a) Bee‐pollinated species from entirely bee‐pollinated tribes (blue) occur, on average, at 653 m, while bee‐pollinated species from tribes containing pollinator shifts (pink) occur, on average, at 820 m. (b) Comparison of Whittaker biome (Whittaker, 1975) occupation of bee‐pollinated species from shifted tribes (pink) and from nonshifted tribes (blue), showing that species from entirely bee‐pollinated tribes mostly occur in tropical rainforests (Biome 5) and savannahs (Biome 6), while bee‐pollinated species from shifted tribes are more common in temperate rainforests (Biome 4). Plot of standardized residuals of the chi‐squared test. Blue indicates a positive correlation, while red indicates a negative correlation. Size of the circles indicates the magnitude of the correlation; faded and small circles indicate a weak correlation. Colored surfaces indicate different biomes, defined by temperature (*x*‐axis) and precipitation (*y*‐axis). (c) Comparison of the annual mean temperature (°C) of bee‐pollinated species from entirely bee‐pollinated tribes and shifted tribes below 1000 m, showing that entirely bee‐pollinated tribes occupy warmer niches in lowlands. (d) Comparison of the annual mean temperature (°C) of bee‐pollinated species from entirely bee‐pollinated tribes and shifted tribes above 1000 m, showing that entirely bee‐pollinated tribes occupy colder niches in mountains. (e) Comparison of the annual precipitation (mm) of bee‐pollinated species from entirely bee‐pollinated tribes and shifted tribes below 1000 m, showing that entirely bee‐pollinated tribes occupy drier niches in lowlands. (f) Comparison of the annual precipitation (mm) of bee‐pollinated species from entirely bee‐pollinated tribes and shifted tribes above 1000 m, showing that entirely bee‐pollinated tribes occupy drier niches in mountains. Boxes represent the interquartile ranges; bars inside the boxes represent the median values; whiskers represent the range; dots represent outliers. * indicate significant differences. *P* values were obtained using phylogenetic *t*‐test. Whittaker biomes: 1, Tundra; 2, Boreal forest; 3, Temperate seasonal forest; 4, Temperate rain forest; 5, Tropical rain forest; 6, Tropical seasonal forest/savanna; 7, Subtropical desert; 8, Temperate grassland/desert; 9, Woodland/shrubland.

To further establish whether bee‐pollinated species from shifted tribes are more likely to occur in environments conducive to pollinator shifts (i.e. mountains) than bee‐pollinated species from nonshifted tribes, we next focused on bee‐pollinated species only (*n* = 280). We found that bee‐pollinated species from tribes containing shifted lineages occur at significantly higher elevations than bee‐pollinated species from nonshifted tribes (phylogenetic mean difference = 772.71, *P* = 1.1e−57; Fig. 4a; Table S6). Species from tribes lacking pollinator shifts did not commonly reach the elevational threshold (> 1000 m) generally triggering pollinator shifts. In line with this, we found bee‐pollinated species of tribes containing pollinator shifts in significantly different biomes (cooler temperate rainforests) than bee‐pollinated species from nonshifted tribes (tropical rain forests and tropical seasonal forests; χ^2^ = 13.51, *P* = 0.009; Fig. 4b).

To explore in more detail whether bee‐pollinated species from shifted and nonshifted tribes occur in different environments at different elevations, we split all bee‐pollinated species into species occurring below the general elevational threshold for pollinator shifts (< 1000 m, *n* = 190) and above (> 1000 m, *n* = 90). Below 1000 m, bee‐pollinated species from shifted tribes occurred in warmer (phylogenetic mean difference = 23.25, *P* = 1.9e−176; Table S7) but wetter (phylogenetic mean difference = 2175.14, *P* = 4.5e−107; Table S8) niches than bee‐pollinated species from nonshifted tribes (Fig. 4c,e). Above 1000 m, however, bee‐pollinated species from shifted tribes occurred in colder (phylogenetic mean difference = 17.73, *P* = 1.3e−37; Table S9) and wetter (phylogenetic mean difference = 1853.53, *P* = 4.2e−43; Table S10) environments than bee‐pollinated species from nonshifted tribes (Fig. 4d,f). These results suggest that bee‐pollinated species from shifted tribes explore a wider range of climatic niche space (generally wetter, but both warmer and colder environments), making it more likely for these bee‐pollinated species to encounter environmental conditions selecting for pollinator shifts.

### Bee‐pollinated species occur in more bee‐friendly environments than shifted relatives

To investigate why mountain environments did not always trigger pollinator shifts, but a substantial proportion of Melastomataceae species retained ancestral bee pollination in mountains, we next tested whether bee‐pollinated montane species are confined to more bee‐friendly (warmer and drier) environments than their vertebrate‐pollinated relatives. Subsetting the dataset to only include montane species (> 1000 m, *n* = 128), we tested how elevation, latitude and precipitation affect the pollination system using binaryPGLMMs. We only retained mean annual temperature after model selection (Tables S11, S12) and found that bee‐pollinated montane species occurred in significantly warmer, but not drier, mountain environments than shifted species (*P* = 4.6−e05; Figs S2b, S5a–d).

### Montane bee‐pollinated species have larger flowers and pores than lowland species

**Fig. 5:**
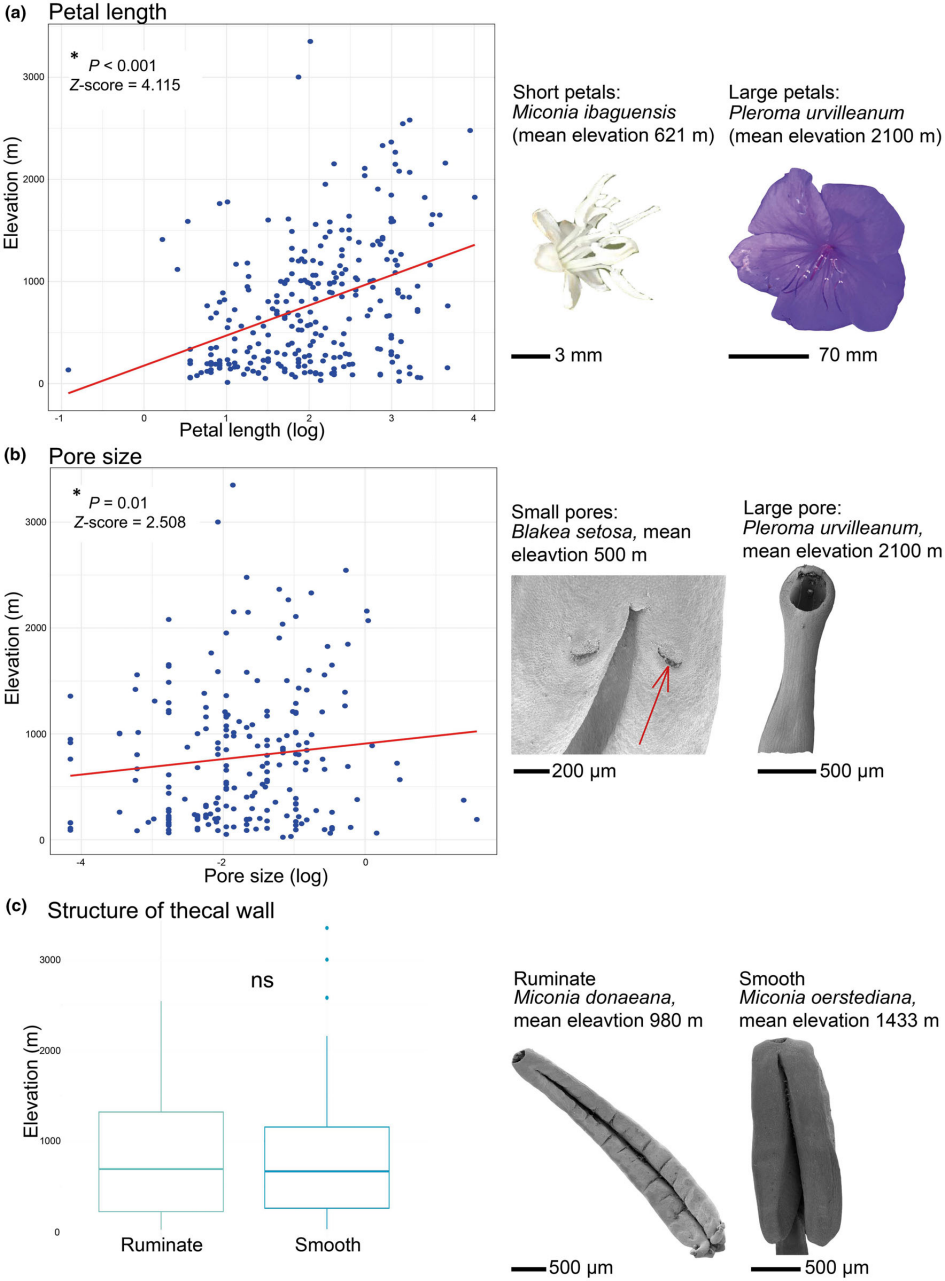
Montane bee‐pollinated Melastomataceae evolved larger flowers and pores (potentially facilitating pollen dispersal) than lowland bee‐pollinated Melastomataceae, but thecal wall structures showed no association with elevation. (a) Scatterplot of petal size on the *x*‐axis and elevation on the *y*‐axis of bee‐pollinated species. (b) Scatterplot of pore size (total pore area: π × ½ pore height × ½ pore width) on the *x*‐axis and elevation on the *y*‐axis. Red line is the regression line of the model (c) Boxplot of elevation data comparing ruminate and smooth anther wall structures of bee‐pollinated species, with no significant difference among the two groups (ns). Boxes represent the interquartile ranges; bars inside the boxes represent the median values; whiskers represent the range and dots represent outliers. *P* values and *Z*‐scores were obtained from the phylogenetic generalized linear mixed models (PGLMM), the raw data are shown in plots.

Among bee‐pollinated species, we tested whether floral traits potentially increasing attractiveness to pollinators (larger flowers) and facilitating pollen dispersal (smooth anther walls and enlarged pores) are more common among species occurring under environmental conditions unfavorable to bee pollination (i.e. high elevations and high precipitation). Indeed, we found that montane bee‐pollinated species have larger flowers (Fig. 5a; residual variance = 0.38, *P*_elevation_ = 3.8e−05 and *P*_latitude_ = 0.01; Tables S13, S14) and pores (Fig. 5b; residual variance = 0.83, *P*_elevation_ = 0.01 and *P*_latitude_ = 0.002; Tables S15, S16), with the best‐fit PGLMM, including elevation and the interaction between elevation and latitude, but not precipitation (Tables S14, S16; Figs 5a,b, S6–S8). At higher latitudes, where elevation zones are naturally depressed, increases in flower and pore size start at lower elevations (Fig. S8). Importantly, we also found that pore size and petal size are uncorrelated (*R* ^2^ = 0.15), indicating independent adaptive evolution of both traits. We did not find any effect of elevation, latitude or precipitation on the distribution of smooth vs corrugated stamen walls (Table S17; Figs 5c, S2c).

## Discussion

Our assessment of the environmental context of pollinator shifts in the large flowering plant family Melastomataceae revealed that evolutionary shifts away from bee pollination have repeatedly been triggered when bee‐pollinated species colonized cool and wet mountain environments (Figs 1a, 3). These shifts were often followed by subsequent niche expansion into even colder and wetter niches. In mountains, bee‐pollinated relatives of shifted species occur in significantly colder and wetter environments than bee‐pollinated species of tribes lacking shifts. This difference in niche occupation among bee‐pollinated species indicates that these cool and wet environments are likely conducive to evolutionary shifts away from bee pollination. Our results hence support the rarely tested hypothesis that certain environmental conditions will consistently trigger pollinator shifts (Stebbins, 1990). Adaptive evolution of flower traits potentially facilitating pollinator attraction (flower size) and pollen dispersal (pore size) may further be critical in enabling some species to retain bee pollination even in environments conducive to pollinator shifts.

### Shifts away from bee pollination associate with cool montane environments

Across Melastomataceae, species which shifted away from ancestral bee pollination mostly occur in montane environments above 1000 m (Figs 2, 3, S9). This pattern matches well with expectations from empirical studies documenting that the harsher climatic conditions prevalent in mountains significantly reduce the abundance and diversity of bee pollinators (Cruden, 1972; Brito & Sazima, 2012; McCallum *et al*., 2013; Classen *et al*., 2015, 2020; Cozien *et al*., 2019). Our results hence support a pollinator‐shift scenario proposed by Thomson & Wilson (2008), which predicts that reductions of bee pollinator visitation rates to flowers may disrupt the stable interaction between plants and their ancestral pollinators (buzzing bees in Melastomataceae) and open the opportunity for pollinator shifts.

The mountain‐pollinator‐shift association reported by us is not only replicated across Melastomataceae but also found across a large sample of Neotropical plant clades, where bee‐pollinated species commonly occur at low elevations and vertebrate‐pollinated relatives occur in montane environments, such as the Andes mountains (e.g. Bromeliaceae, Gesneriaceae, *Psychotria*, *Passiflora*, Loranthaceae and *Salvia*; Dellinger *et al*., 2023). While the lack of detailed assessments of the relative timing of mountain colonization and pollinator shifts in these clades currently impedes inference of causality, the increased abundance of vertebrate (particularly hummingbird) pollination in tropical mountains points toward an overwhelming role of the abiotic environment in triggering pollinator shifts (i.e. ‘environments conducive to evolutionary pollinator shifts’, compare to the idea of ‘hummingbird habitats’ by Stebbins, 1990). Importantly, such ‘environments conducive to evolutionary pollinator shifts’ might be clade‐specific or depend on the respective biogeographic context. For example, lower elevations associate with shifts to hummingbird pollination in temperate *Penstemon* (while bee‐pollinated species occur at higher elevations, Hamilton & Wessinger, 2022). Also, in some clades, pollinator shifts seemingly occur in the absence of environment shifts (i.e. Melastomataceae tribe Astroniaeae; Figs 3b, S3b). The latter is true also for the Neotropical section of the genus *Costus*, where pollinator shifts are unrelated to elevation (Kay & Grossenbacher, 2022). In such cases, alternative mechanisms, such as competition for pollinators or major mutations in floral traits involved in pollinator attraction (i.e. color and scent), may lead to pollinator shifts even without ecogeographic divergence (Bradshaw & Schemske, 2003; Hoballah *et al*., 2007; Muchhala & Potts, 2007; Peakall *et al*., 2010; Xu *et al*., 2011; Binaghi *et al*., 2023). Investigating the link between pollinator shifts and environment shifts across more plant clades and biogeographic contexts in the future is necessary to establish whether environment‐induced reduction in the abundance and efficiency of ancestral pollinators is indeed a common driver of evolutionary pollinator shifts.

Another aspect worth investigating in more detail is whether each functional group of pollinators has its respective ‘environment conducive to shifts’ (i.e. where its abundance and efficiency is significantly reduced in comparison with other groups). We here merged all species belonging to shifted pollination syndromes (‘generalist’, ‘nectar‐foraging vertebrate’, ‘food‐body‐foraging vertebrate’) to broadly evaluate whether shifts away from bee pollination are consistently triggered by the same environmental variables. Among the shifted melastome groups, however, ‘generalist’ syndrome Melastomataceae are noteworthy as occurring at lower elevations (even at lower latitudes) than vertebrate‐pollinated species (Fig. 2c,d). This trend may be due to undersampling of comparatively inconspicuous generalist Miconieae in the Andes, with numerous recent reports of generalist pollination in *Miconia* in the tropical Andes (Valderrama *et al*., 2022, Angulo *et al*., unpublished). Alternatively, there may be conditions currently unknown that favor transitions to generalist flowers in Melastomataceae, which, being smaller in size with lower amounts of nectar (Kopper *et al*., 2024), may be cheaper to produce than vertebrate‐adapted flowers.

In addition, environments conducive to pollinator shifts may not always be geographical, but also seasonal, with different seasons favoring different reproductive/pollination strategies. For example, Brito *et al*. (2017) found that pollinator‐dependent bee‐pollinated Melastomataceae flowered during the warmer rainy season and pollinator‐independent (autogamous self‐pollinating and apomictic) species flowered during the colder dry season in the Brazilian Atlantic Forest. We could thus envision scenarios in Melastomataceae where divergence in flowering times (i.e. due to pollinator competition) from the warm ‘bee‐friendly’ to the cold ‘shift’ season could trigger pollinator shifts independent of elevation. Such seasonality‐linked pollinator shifts have been reported for South African orchids (Peter & Johnson, 2014) and are also known from Asia and Africa, where vertebrate‐pollinated species tend to flower in the cold and rainy season (Janeček *et al*., 2015; Funamoto, 2019). Again, these season‐shift links underscore the importance of the abiotic environment in understanding the evolution of pollinator shifts.

### Colonization of mountain habitats precedes pollinator shifts

Our finding that entire Melastomataceae clades shift into mountains typically before pollinator shifts occur (Fig. 3; Table 1) supports the idea that niche expansion into environments conducive to pollinator shifts preceded pollinator shifts. Most species with empirically verified pollinator shifts in our sample occur in geologically relatively young mountain ranges with final phases of mountain uplift during the mid/late Miocene (15–5 Million years ago; tropical Andes (Boschman & Condamine, 2022), New Guinea (Roycroft *et al*., 2022), East African Mountains (Dellinger *et al*., 2022; Dagallier *et al.*, 2024; but also ancient Western Ghats, Johnson *et al*., 2022)). Accordingly, our reconstruction of the elevational history of Melastomataceae shows repeated colonization of these uplifting mountains within the past 20–30 Myr, followed by shifts away from bee pollination commonly only within the past 10 Myr (Table 1). A scenario thus seems plausible where bee‐pollinated ancestors of present‐day Melastomataceae colonized the newly forming mountain ranges from lowland rainforests. With the generally reduced availability of bee pollinators in these mountain environments (Arroyo *et al*., 1982; Classen *et al*., 2020), successive niche filling might then have intensified competition for the already limited resource of montane bee pollinators, spurring evolutionary shifts to vertebrate pollination and generalists (Armbruster, 1985). Clearly, the interpretation of our analyses is affected by how elevation is defined, with clearest support for environment shifts preceding pollinator shifts when classifying species > 500 m as montane (which captures latitudinal differences, generalist species and island effects). Since the general trend that tribe crown nodes occur at lower elevations than shifted nodes (Table 1) holds irrespective of binarizing elevation, we are confident that environment shifts are indeed a precursor to pollinator shifts in most Melastomataceae lineages.

Another result revealed by our analyses is that lineages containing species which shifted pollinators diversified into even colder environments and higher elevations than their bee‐pollinated ancestors and relatives (Fig. 3). Thus, while mountains likely provided the initial environments conducive to pollination shifts, shifts to efficient high‐elevation pollinators, such as hummingbirds, sunbirds and white‐eyes, allowed for subsequent niche expansion to even higher elevations in tropical mountains. At least in Neotropical mountains, this expansion into higher elevations may also represent ‘niche tracking’ of the newly acquired hummingbird pollinators, which concomitantly diversified in the cold and wet cloud forests during the Miocene (Barreto *et al*., 2023).

### Bee‐pollinated species of shifted tribes occur in environments conducive to pollinator shifts

We found that bee‐pollinated species of tribes containing pollinator shifts generally occur in wetter niches compared with bee‐pollinated species from tribes without pollinator shifts (Fig. 4f). Bee‐pollinated montane species from shifted tribes are hence more likely to be exposed to environments conducive to pollinator shifts (i.e. cold and wet conditions reducing bee flower visitation activity) than species from nonshifted tribes. Bee‐pollinated species from tribes lacking pollinator shifts seem to have only colonized warmer and drier subniches in mountains (i.e. rain‐sheltered slopes). Whether these niche differences among bee‐pollinated species from shifted and nonshifted tribes are caused by physiological constraints (i.e. limited temperature tolerance; Eller *et al*., 2020), genetic constraints (i.e. inability to evolve certain traits, Bradshaw, 1991), biogeographic history (i.e. ancestral presence in drier mountains), dispersal limitations (i.e. remaining on drier slopes; Polato *et al*., 2018) or lower adaptability to montane bee pollinators (to be described later), remains to be explored.

### Montane bee‐pollinated species occur in more bee‐friendly environments than shifted relatives

Contrasting bee‐pollinated and shifted species found above 1000 m, we found that bee‐pollinated species grow in warmer environments than their shifted relatives (Fig. S5b). These warmer montane environments may be more ‘bee friendly’, featuring higher bee diversity and abundance and thus bee pollinator availability (Brito & Sazima, 2012; Dellinger *et al*., 2021). Melastomataceae species colonizing such ‘bee‐friendly’ environments in tropical mountains might thus not have experienced the radical reduction in bee pollinator abundance otherwise triggering pollinator shifts, thus maintaining the stable buzz pollination system.

An aspect that we could not assess in this study due to a lack of detailed bee pollinator data is how turnovers in the functional (i.e. body size) and taxonomic composition of bee pollinator assemblages across elevation affect pollinator shifts. Bee body size, for example, generally determines the thermoregulation capacity of bees, with larger bees being able to maintain high body temperatures more efficiently even in low temperature environments (Hodkinson, 2005). Accordingly, bee body size commonly increases along elevational gradients and often is paralleled by a narrowing in bee taxonomic diversity to relatively large‐bodied genera (e.g. *Bombus*, *Eufriesea* and *Centris*; McCabe *et al*., 2019). Furthermore, some bee families, such as buzz‐pollinating Euglossini, show marked latitudinal differences, with large‐bodied genera, such as *Eulaema*, *Eufriesea* and *Exaerete*, common both in the Andes and Amazonian lowlands, but small‐bodied *Euglossa* species are more common in Central America (Roubik & Hanson, 2004; Kay & Grossenbacher, 2022). Collecting more detailed data on Melastomataceae bee pollinator communities will be essential in the future to test whether montane bee‐pollinated Melastomataceae are generally adapted to pollination by larger buzzing bees than lowland bee‐pollinated Melastomataceae. It is possible that shifts to larger bee pollinators facilitated mountain colonization of Melastomataceae and that bee‐pollinated species from shifted clades are generally adapted to bees with more extreme (i.e. cold) environmental tolerances.

### Montane bee‐pollinated species have larger flowers and anther pores than lowland species

Besides pollinator shifts, adaptive trait evolution may mitigate negative effects of reduced pollinator availability. Accordingly, among montane Melastomataceae, we found an increase in petal length and anther pore size (Fig. 5). Larger floral display is known to increase pollinator attraction (Dafni *et al*., 1997; Goodwillie *et al*., 2010) and may increase a species' probability of being visited. In addition, species may adapt their pollen dosing to the abundance and efficiency of their pollinators and release pollen in larger portions if pollinator visits are rare (Castellanos *et al*., 2006). Both mathematical models (Boucher‐Bergstedt *et al*., 2025) and experiments on the Melastomataceae species *Adelobotrys adscendens* (Sw.) Triana (Dellinger *et al*., 2019a) suggest that larger pores may indeed release larger proportions of pollen. The increase in anther pore size with elevation in Melastomataceae may thus represent an adaptation to release larger quantities of pollen with the infrequent bee visits.

With the general increase in bee pollinator body size at higher elevations, we cannot rule out that the increase in flower and pore size is merely related to the larger pollinator size (Classen *et al*., 2015) rather than a mechanism to optimize pollen dispersal. The fact that flower and pore size are uncorrelated, however, suggests that the two traits may underlie different selection pressures so that selection for relaxed pollen dosing may indeed play a significant role in montane bee‐pollinated Melastomataceae. Clearly, extensive comparative experiments on floral biomechanics are required to clarify the adaptive potential of flower and pore size. If functional relationships are indeed revealed, the evolution of increased floral display and enlarged anther pores may have been critical to allow for the initial colonization of montane environments by Melastomataceae, as well as for the persistence of bee‐pollinated species in tropical mountains.

### Conclusion

Although evolutionary shifts among functional groups of pollinators are commonly recognized as a key process in angiosperm diversification, our current understanding of the mechanisms triggering pollinator shifts is limited. In this study, using the tropical plant family Melastomataceae as a model, we investigated the role of environmental factors in explaining pollinator shifts and found that climatic niche shifts (into mountain environments) acted as important precursors for evolutionary shifts away from bee pollination. Specifically, Melastomataceae showed a pattern common for Neotropical plants, where shifts away from bee pollination associate with occurrence in montane environments (Dellinger *et al*., 2023). These macroevolutionary associations confirm empirical case studies (Cruden, 1972; Dellinger *et al*., 2021), suggesting that the reduction in bee pollinator abundance induced by montane climatic conditions acts as an important trigger of pollinator shifts (Thomson & Wilson, 2008). Our results raise the question of whether climatic niche shifts followed by pollinator shifts are a common pattern in angiosperm diversification (Donoghue & Sanderson, 2015). More broadly, such staggered evolutionary events and strong linkage of ecogeographic patterns and biotic interactions may transcend beyond plant–pollinator interactions and be characteristic also for other types of plant–animal interactions, such as ant–plant mutualisms (Luo *et al*., 2023) or seed dispersal (Salazar‐Tortosa *et al*., 2019). Investigating evolutionary dynamics between climatic niche shifts and shifts in biotic interactions across a diversity of interaction types and plant clades represents a major avenue for future research.

## Competing interests

None declared.

## Author contributions

CK, JS and ASD conceived the study. CK compiled the data matrix. CK and ASD carried out the statistical analyses. CK wrote the manuscript. All authors contributed to finalizing the manuscript. JS and ASD contributed equally to this work and joint senior authors.

## Disclaimer

The New Phytologist Foundation remains neutral with regard to jurisdictional claims in maps and in any institutional affiliations.

## Supporting information


**Fig. S1** Correlation plots of climatic variables and elevation and distribution maps of pollination system.
**Fig. S2** Reconstruction of pollination systems and elevation.
**Fig. S3** Model fit of binaryPGLMM models.
**Fig. S4** Ornstein–Uhlenbeck models for elevational optima.
**Fig. S5** Bee‐pollinated species generally occur in warmer environments than vertebrate‐pollinated.
**Fig. S6** Model fit of PGLMM for petal length.
**Fig. S7** Model fit of PGLMM for pore size.
**Fig. S8** With increasing latitude, petal and pore size of bee‐pollinated species start to increase already at lower elevation.
**Fig. S9** Tip states of elevation and pollination system mapped on a phylogeny (Reginato *et al*., 2022).
**Notes S1** Methods SI.
**Notes S2** Climatic variables.

**Table S1** GBIF occurrence points of the 333 species included in this study.

**Table S2** AIC of MuSSE models.
**Table S3** BinaryPGLMM full model for pollination syndromes using elevation, latitude and annual precipitation.
**Table S4** BinaryPGLMM reduced model for pollination system using elevation and latitude.
**Table S5** pBIC of OU models testing for elevational optima.
**Table S6** Phylogenetic *t*‐test using elevation.
**Table S7** Phylogenetic *t*‐test for bee‐pollinated species from shifted and nonshifted tribes below 1000 m using annual mean temperature.
**Table S8** Phylogenetic *t*‐test for bee‐pollinated species from shifted and nonshifted tribes below 1000 m using annual precipitation.
**Table S9** Phylogenetic *t*‐test for bee‐pollinated species from shifted and nonshifted tribes above 1000 m using annual mean temperature.
**Table S10** Phylogenetic *t*‐test for bee‐pollinated species from shifted and nonshifted tribes above 1000 m using annual precipitation.
**Table S11** BinaryPGLMM full model for pollination system above 1000 m using annual mean temperature and annual precipitation.
**Table S12** BinaryPGLMM reduced model for pollination system above 1000 m using annual mean temperature.
**Table S13** PGLMM full model for petal length of bee‐pollinated species using elevation, latitude and annual precipitation.
**Table S14** PGLMM reduced model for petal length of bee‐pollinated species using elevation and latitude.
**Table S15** PGLMM full model for pore size of bee‐pollinated species using elevation, latitude and annual precipitation.
**Table S16** PGLMM reduced model for pore size of bee‐pollinated species using elevation and latitude.
**Table S17** BinaryPGLMM full model for structure of thecal wall using elevation, latitude and annual precipitation.
Please note: Wiley is not responsible for the content or functionality of any Supporting Information supplied by the authors. Any queries (other than missing material) should be directed to the *New Phytologist* Central Office.

## Data Availability

All datasets, R‐codes and the floral trait matrix have been deposited in the open access repository phaidra (https://phaidra.univie.ac.at/o:2096899).
