# Supplementary material for: Mountain colonization precedes shifts away from bee pollination in Melastomataceae
Source: New Phytol. 2025 Jun 8;247(3):1474–92. doi: 10.1111/nph.70273 (PMC12222925; doi:10.1111/nph.70273)
Supplement: Supplementary file 1 — Fig. S1 Correlation plots of climatic variables and elevation and distribution maps of pollination system. Fig. S2 Reconstruction of pollination systems and elevation. Fig. S3 Model fit of binaryPGLMM models. Fig. S4 Ornstein–Uhlenbeck models for elevational optima. Fig. S5 Bee‐pollinated species generally occur in warmer environments than vertebrate‐pollinated. Fig. S6 Model fit of PGLMM for petal length. Fig. S7 Model fit of PGLMM for pore size. Fig. S8 With increasing latitude, petal and pore size of bee‐pollinated species start to increase already at lower elevation. Fig. S9 Tip states of elevation and pollination system mapped on a phylogeny (Reginato et al., 2022). Notes S1 Methods SI. Notes S2 Climatic variables. [file NPH-247-1474-s003.pdf]

New Phytologist **Supporting Information**

Article title: Mountain colonization precedes shifts away from bee pollination in Melastomataceae
Authors: Constantin Kopper, Jürg Schönenberger, Agnes S. Dellinger
Article acceptance date: 14 May 2025

The following Supporting Information is available for this article:

Fig. S1 Correlation plots of climatic variables and elevation and distribution maps of pollination system. (a) Correlation plots; Bio1 = Annual Mean Temperature, Bio12 = Annual Precipitation; red indicates a negative correlation while blue indicates a positive correlation; the size of the circle and its color intensity indicate how strong a correlation is. (b) Distribution maps of pollination system.

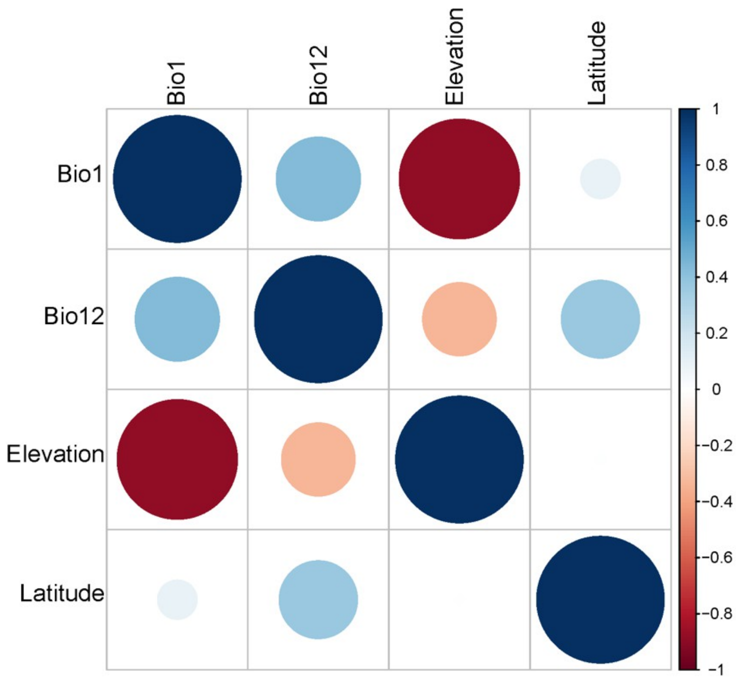

a

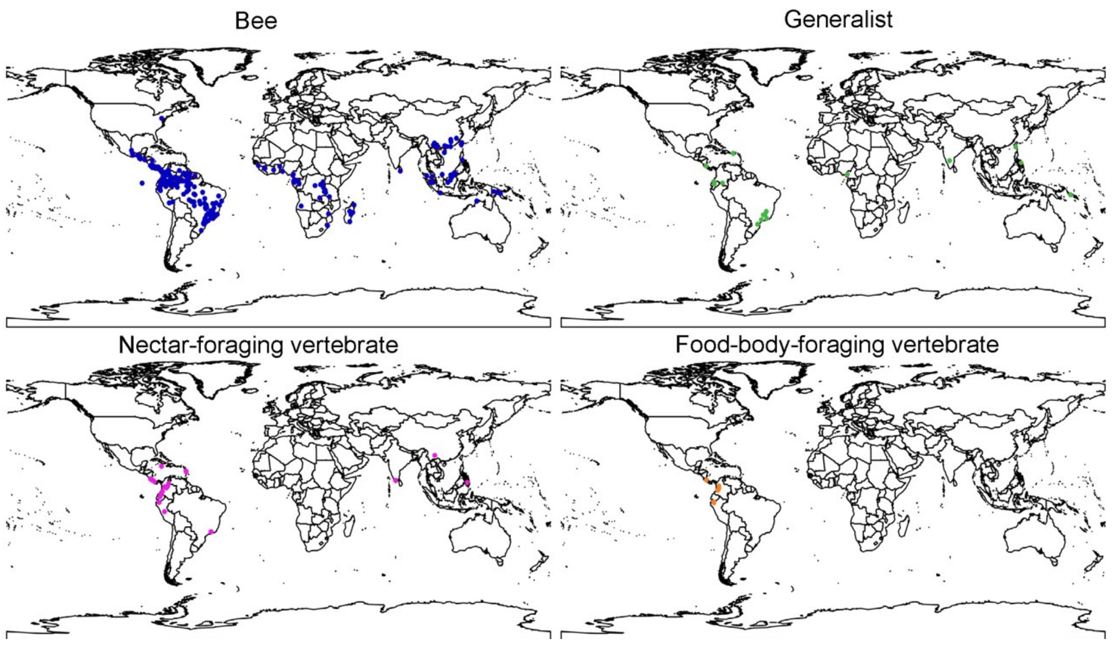

Fig. S2 Model fit of binaryPGLMM models. (a) binaryPGLMM with stepwise variable selection modeling the impact of elevation, latitude, annual precipitation (Bio12, M1), of which elevation and latitude showed a significant impact on pollination system ($p_{elevatio}$n = 1.2e-08, $p_{latitude}$ = 0.02); (b) binaryPGLMM with stepwise variable selection modeling the impact of annual mean temperature (Bio1) and annual precipitation (Bio12, M2) on pollination system above 1000 m, of which only mean temperature was significant (p = 4.6-e05); (c) binaryPGLMM model for only

bee-pollinated species with stepwise variable selection modeling the impact of elevation, latitude and annual precipitation (Bio12, M1) on thecal wall structure, no variable showed a significant impact on thecal wall structure using only bee-pollinated species (p = 0.12).

Model fit of binaryPGLMM

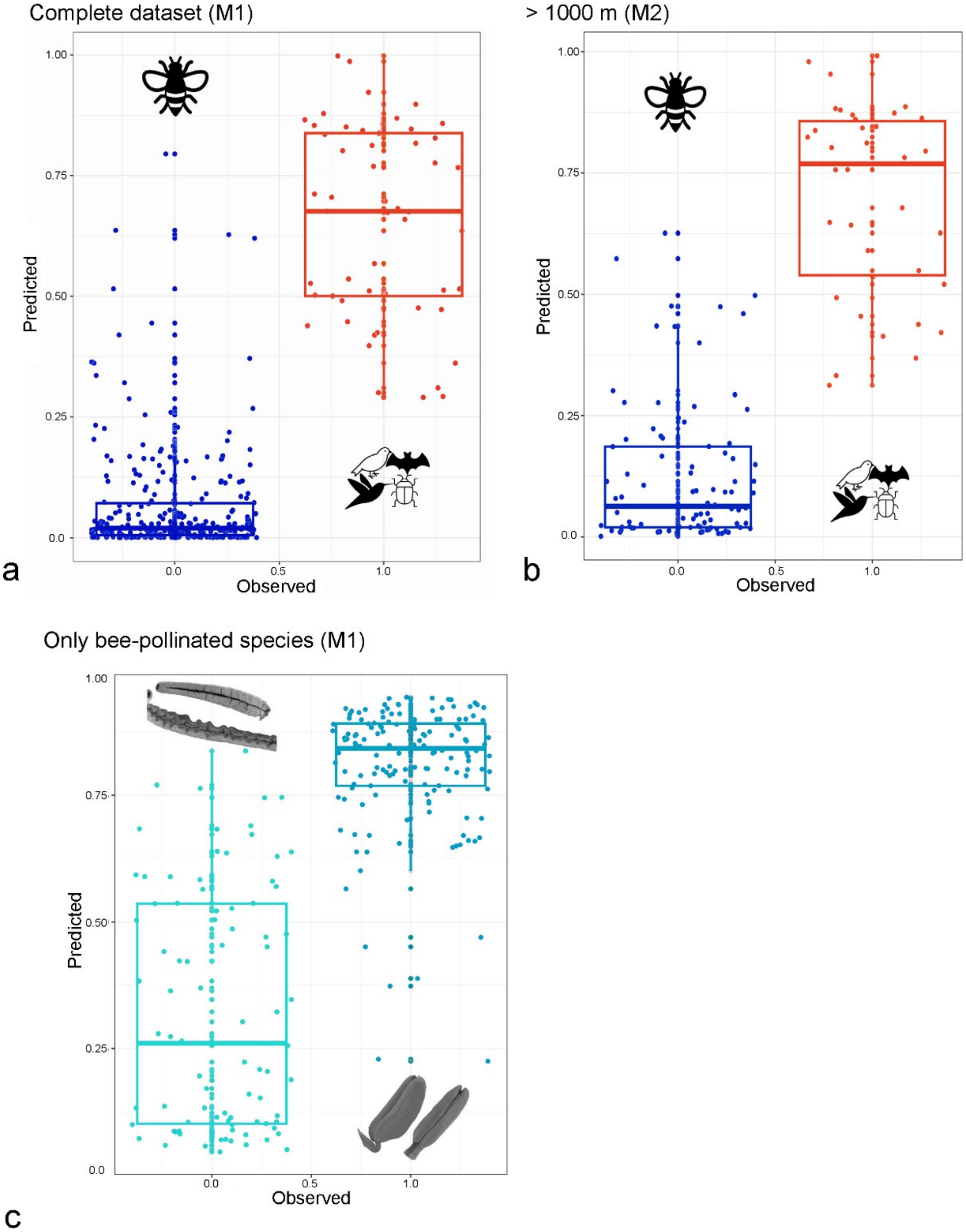

Fig. S3 Reconstruction of pollination systems and elevation. (a) the two dated phylogenetic trees are based on the analysis by Reginato et al. (2022) with the 333 tips matching the taxon sampling of the present study; in the left tree, tip states indicate pollination strategy; pie charts at nodes represent probabilities of different pollination system at that node (blue = bee pollination, orange = shifted pollination system), numbers indicate tribes: 1 = Olisbeoideae, 2 = Kibessieae; 3 = Merianieae, 4 = Miconieae, 5 = Eriocnemeae, 6 = Trioleneae, 7 = Rupestreae, 8 = Rhexieae, 9 = Microlicieae, 10 = Melastomateae, 11 = Marcetieae, 12 = Dinophoreae, 13 = Dissochaeteae, 14 = Cambessedesieae, 15 = Stanmarkieae, 16 = Cyphostyleae, 17 = Sonerileae, 18 = Pyxidantheae, 19 = Bertolonieae, 20 = Lithobieae, 21 = Henrietteeae, 22 = Astronieae, tribes containing species which shifted pollinators: 1, 3, 4, 10, 17, 19, 22; in the right tree, branch colors indicate the elevational gradient (red = low altitude, blue = high altitude). (b) pollinator shifts go hand in hand with shifts in elevation. We found positive slopes going from 100% probability of being bee-pollinated to 100% probability of being shifted in 19 out of the 25 shifts across all clades

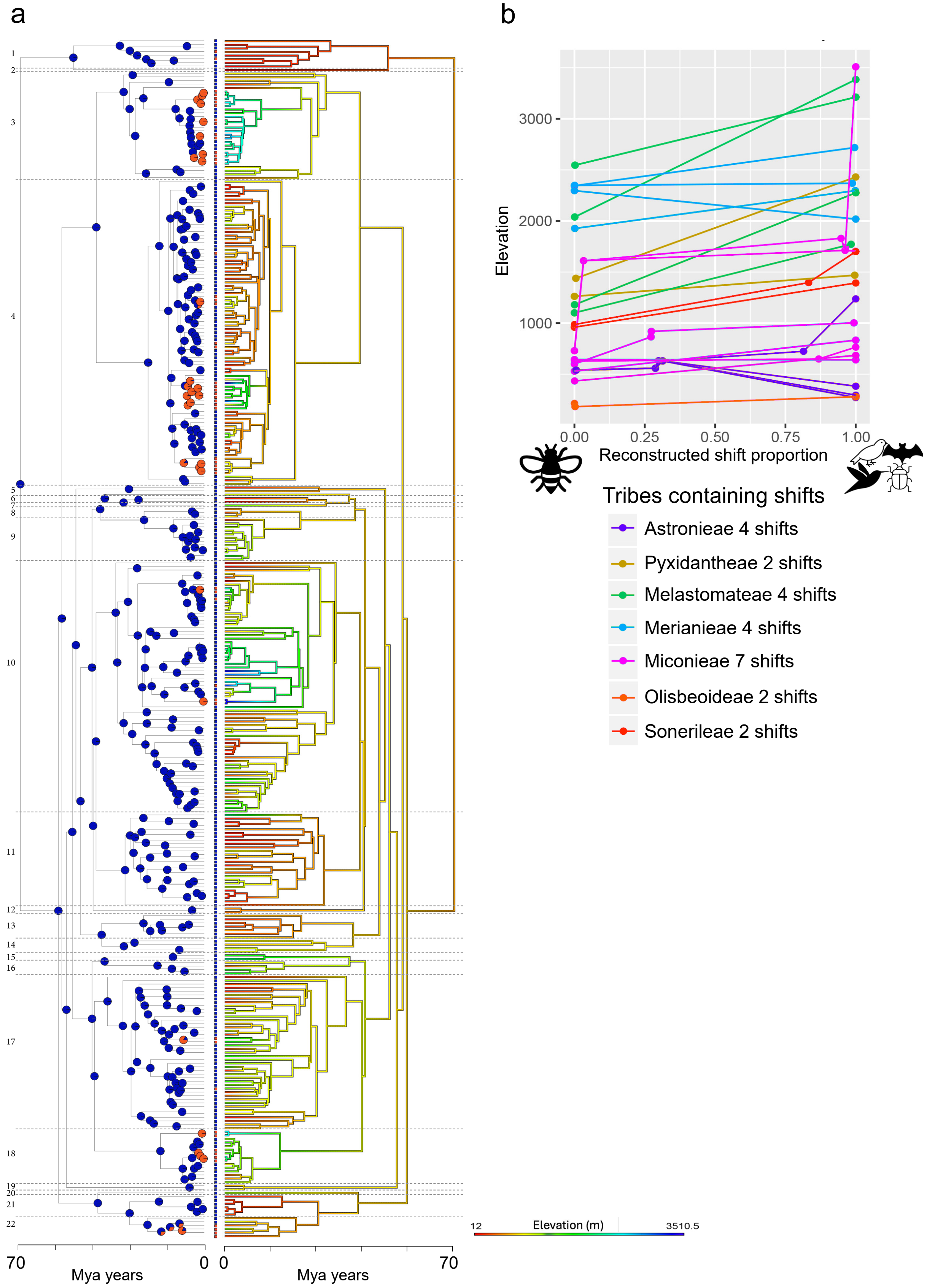

a
b
1
2
3
4
5
6
7
8
9
10
11
12
13
14
15
16
17
18
19
20
21
22
70
Mya years
0
0
Mya years
70
12
Elevation (m)
3510.5
Elevation
3000
2000
1000
0.00
0.25
0.50
0.75
1.00
Reconstructed shift proportion
Tribes containing shifts
Astronieae 4 shifts
Pyxidantheae 2 shifts
Melastomateae 4 shifts
Merianieae 4 shifts
Miconieae 7 shifts
Olisbeoideae 2 shifts
Sonerileae 2 shifts

Fig. S4 Ornstein-Uhlenbeck-Models for elevational optima. (a) Best OU-Model (pBIC = 5310.58) showing elevational optima for tribes containing species which have shifted pollinators. There are three elevational optima: one for the tribe Olisbeoideae (green), one convergent optimum for the tribes Merianieae, Melastomateae, and Pyxidantheae (blue) and one for all other tribes (gray) including three tribes containing species which have shifted pollinators (Miconieae, Sonerileae, Astronieae). (b) Second best OU-Model (pBIC = 5355.78) testing for separate elevational optima for "nectar-foraging vertebrate" together with "food-body-foraging vertebrate" syndrome and "buzz-bee" together with "generalist" syndrome we could show that shifts to the "generalist" syndrome start already at lower elevation than shifts to other pollination systems. There are two convergent elevational optima: ten convergent elevational shifts (blue), three convergent elevational shifts (yellow) and four independent elevational shifts. (c) Third best OU-Model (pBIC = 5451.59) testing for separate elevational optimal for species which have shifted pollinators and those which remained bee-pollinated. There are two convergent elevational optima: eight convergent elevational shifts (yellow), three convergent elevational shifts (green) and three independent elevational shifts.

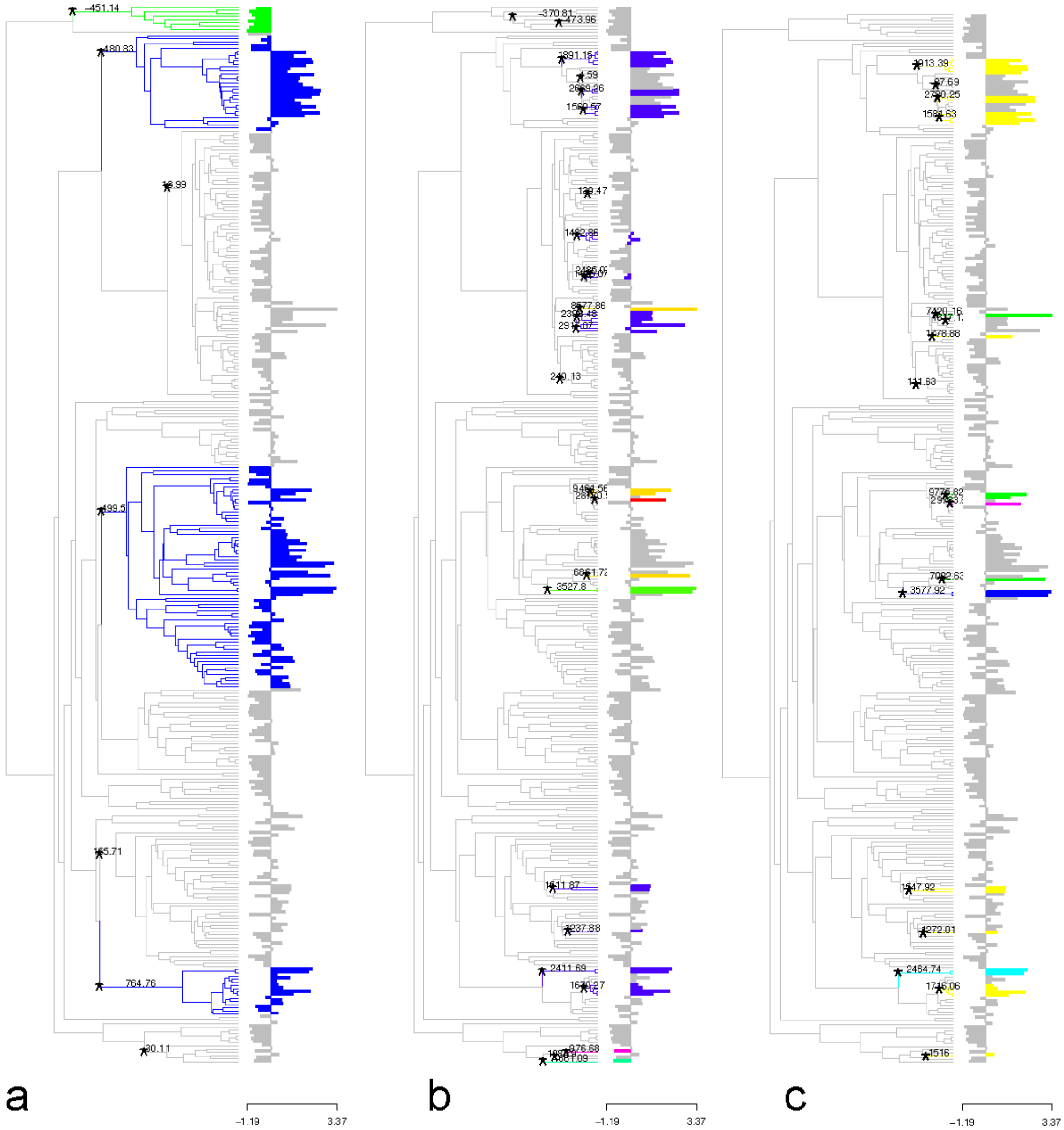

−451.14
480.83
499.5
764.76
80.11
−370.81
473.96
1891.15
2411.69
3527.8
1237.88
1913.39
2464.74
3577.92
1547.92
1272.01
1516
a
b
c
−1.19
3.37
−1.19
3.37
−1.19
3.37

Fig. S5 Bee-pollinated species generally occur in warmer environments than vertebrate-pollinated species (a, b), while there is no difference between the two groups with respect to in annual precipitation (c, d). We calculated corrected p values by using phylANOVA.

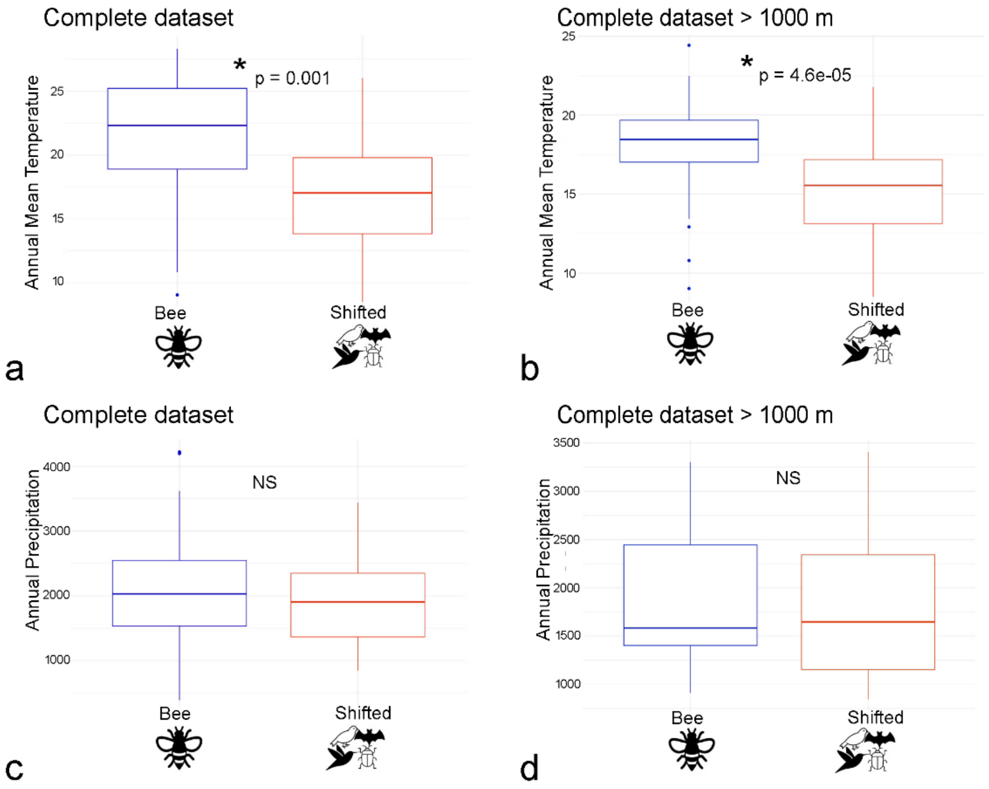

Fig. S6 Model fit of PGLMM for petal length. Showing the observed and the predicted values by the PGLMM model for petal length Each blue point represents an observation. The red line is serving as a reference for perfect prediction, where points along this line would represent ideal model accuracy. Discrepancies between observed and predicted values are visualized by the distance of points from this line

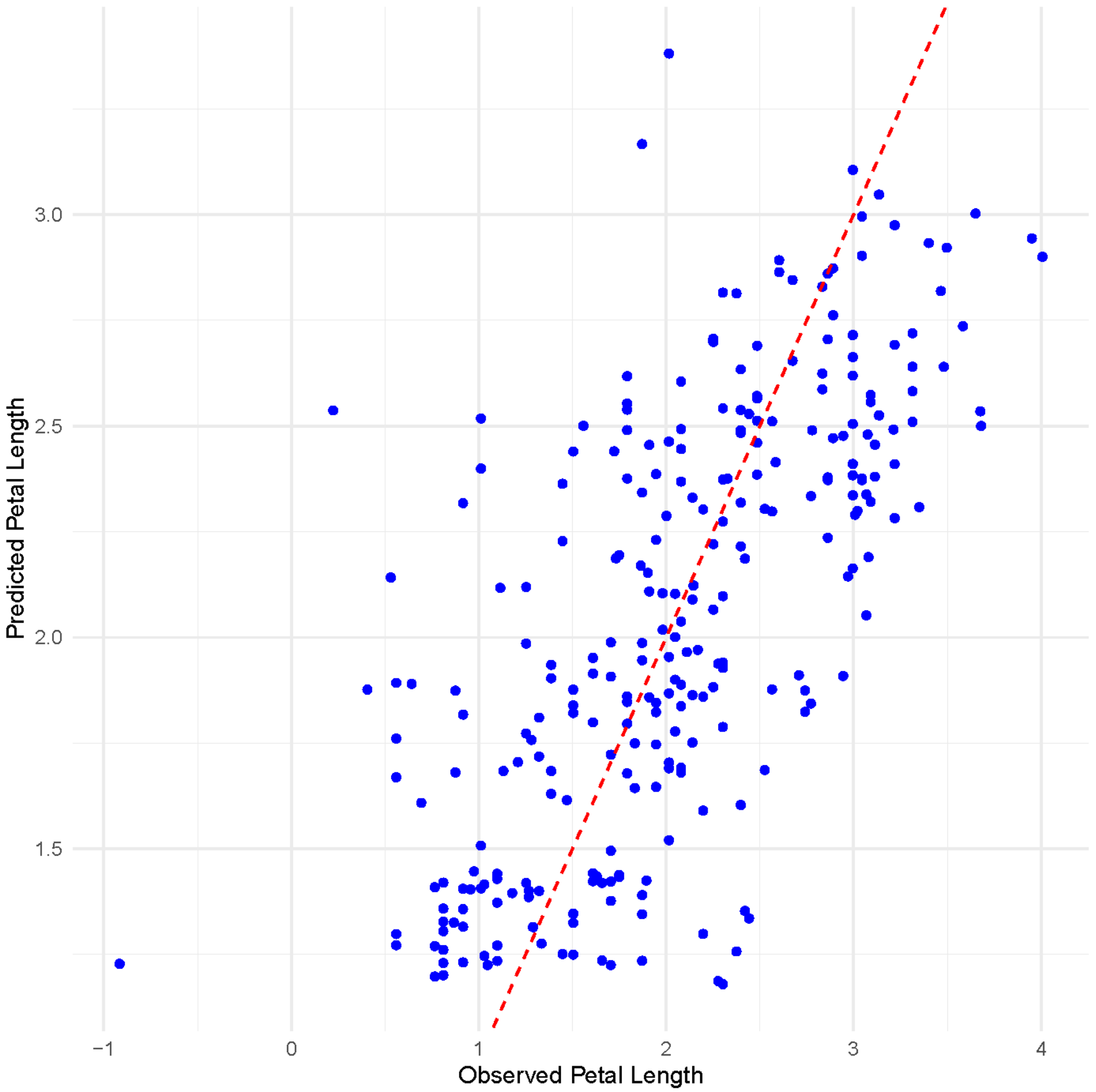

Fig. S7 Model fit of PGLMM for pore size. Showing the observed and the predicted values by the PGLMM model for pore size. Each blue point represents an observation. The red line is serving as a reference for perfect prediction, where points along this line would represent ideal model accuracy. Discrepancies between observed and predicted values are visualized by the distance of points from this line.

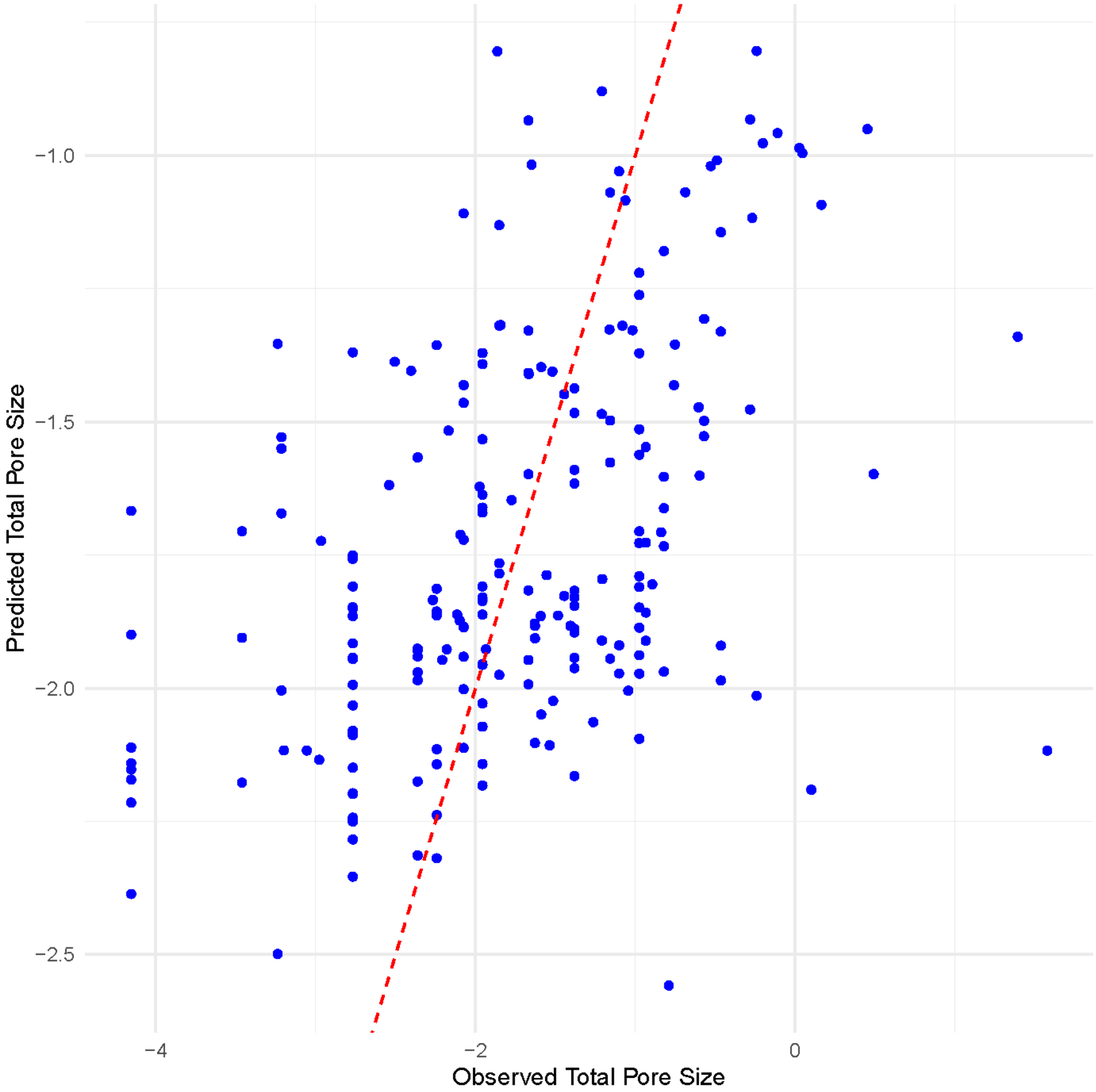

Fig. S8 With increasing latitude, petal- and pore size of bee-pollinated species start to increase already at lower elevation. (a) Pore size of bee-pollinated species increases with increasing elevation; with increasing latitude, pore size increases already at lower elevation. (b) Petal length of bee-pollinated species increases with increasing elevation; with increasing latitude petal size increases already at lower elevation. Blue indicates small values while red indicates larger values. The line on the upper side of the graph represents the number of bee-pollinated species at a given elevation while the line on the right hand side of the graph represents the number of bee-pollinated species at a given latitude.

Pore Size

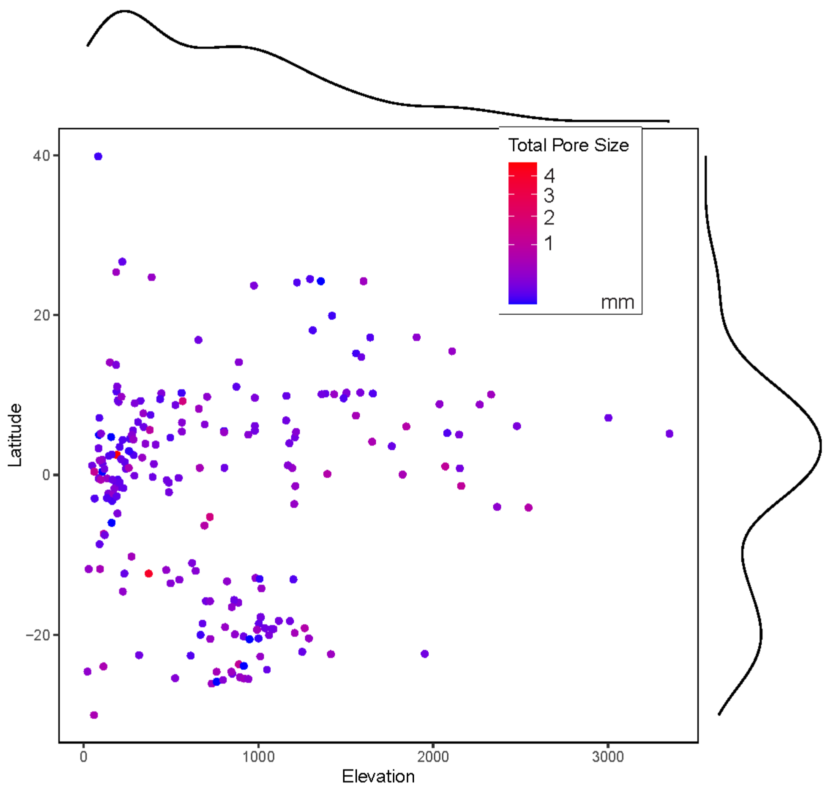

Petal Length

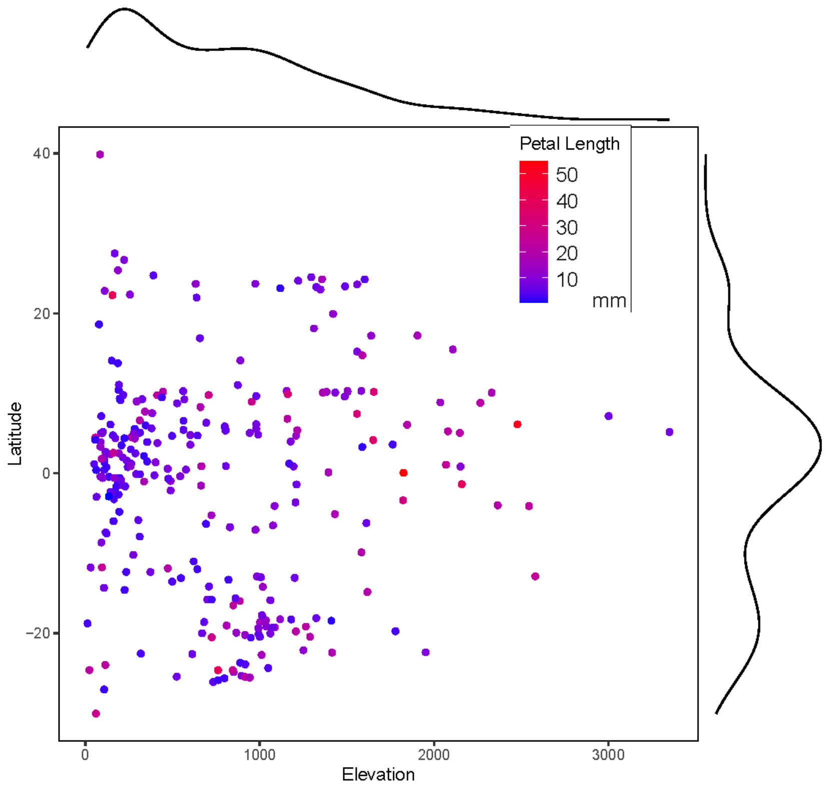

Fig. S9 Tip states of elevation and pollinations system mapped on a phylogeny (Reginato et al. 2022). The buzz-bee syndrome can be found along the whole elevational gradient while most species which have shifted pollinators start to occur at about 1000 m (some species, primarily "generalist" syndrome species, already occur below 1000 m). On the right side of the plot the elevation at which each species occurs is depicted; not that the bars do not show an elevational range, only the tip of the bars represent the represent the elevation species occur at.

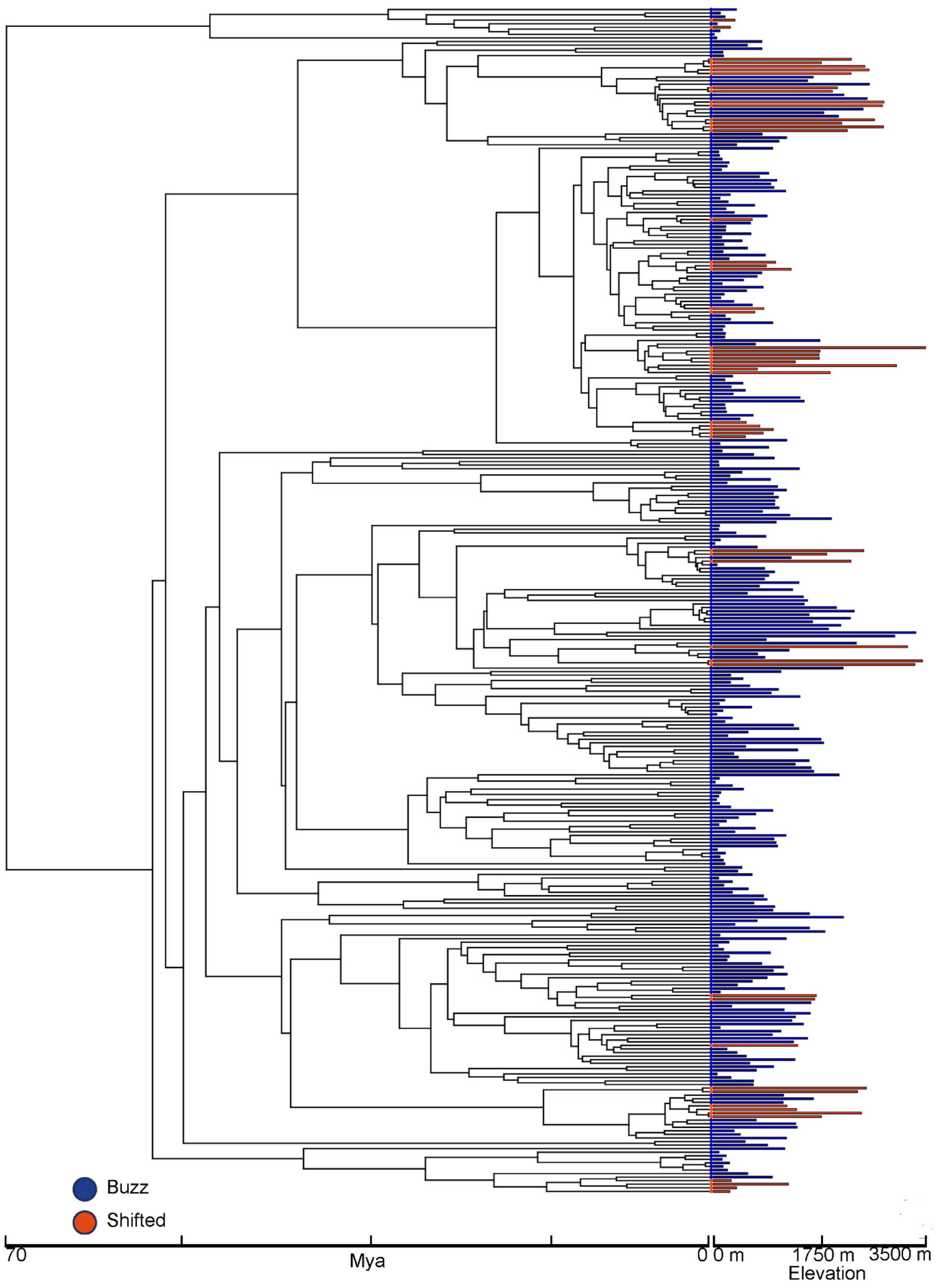

Buzz
Shifted
70
Mya
0
0 m
1750 m
3500 m
Elevation

Notes S1 Material and Methods

We ran all statistical analyses in R (R Core Team 2022).

Species selection, pollination mode and phylogenetic hypothesis

Our analyses were based on a dataset of 333 species (5.79% of Melastomataceae), for 206 of which floral visitors were empirically documented (including visitors that were verified as pollinators or were shown to have at least a high potential to be legitimate pollinators; Dellinger et al. 2022). For the remaining 127, we have predicted pollinators in a previous study using machine learning-based classification methods (Random Forest analyses), where we trained initial models on 44 floral traits and 252 species with documented pollinators. Then we used these trained and validated (high prediction accuracy) models to predict pollinators for the remaining species (Kopper et al. 2024). Of the 333 species used in this study, 53 species across seven tribes have shifted pollinators. These shifted pollination systems can be summarized into three syndromes: "generalist" (flies, wasps, beetles, butterflies, bees), "nectar-foraging vertebrate" (hummingbirds, flower piercers, lizards, rodents, bats) and "food-body-foraging vertebrate" (passerine birds, parrots, Dellinger et al. 2022, Kopper et al. 2024). For our analyses, we merged these three pollination syndromes (all derived from the ancestral "buzz-bee" syndrome) into "shifted" since we wanted to test whether there are general associations between shifts and environmental conditions. Also, this grouping was necessary to assure sufficient evolutionary replication.

We used the most recent dated molecular phylogeny for Melastomataceae (Reginato et al. 2022, 2454 tips, crown node age 68.5 – 75.2 my; based on the ML topology of Penneys et al. 2022). We used the MCC-tree from Reginato et al. (2022) and pruned the tips to the species in our data matrix (*treedata* function, geiger v2.0.11, Alfaro et al. 2009). All tribes except Feliciadamieae were covered by this phylogeny. Except for Sonerileae, at least one shifted species from each tribe containing shifts (Kopper et al. 2024) was included in the phylogeny, a critical pre-condition for accurately estimating the evolution of pollination systems. The shifts in Sonerileae (*Medinilla malabarica* Bedd. & C.E.C.Fisch, *M. sahyadrica* Sujanapal & Sasidh.) missing from the phylogeny stem from the same sub-clade. In order to also represent this sub-clade (and hence the shifts) in our analysis, we selected two close relatives as surrogate tips from the same sub-clade (*Medinilla mannii* Hook.f, *M. micrantha* Jum. & H.Perrier). This choice was based on a currently ongoing, detailed phylogenetic treatment of the genus *Medinilla* (Quakenbush, pers. com.). No bee-pollinated *Medinilla* taxa from the same sub-clade were included, so that these surrogate tips represent an independent pollinator shift and do not confound our analyses.

For subsequent analyses, we grouped species into the currently recognized 23 Melastomataceae tribes based on Penneys et al. (2022). Taxonomy strictly follows phylogenetic principles, i.e., all tribes are monophyletic. Note that we use the term *tribe* throughout the manuscript to refer to these 23 taxonomic tribes, while we use the term *clade* for groups of closely related species *within* a tribe which all share a common ancestor (i.e., shifted and non-shifted clades within tribes).

GBIF occurrence data, elevation, and climatic variables

We screened the initial plant species list (n = 336) using Taxonstand (U.Taxonstand version 2.4, Zhang & Qian 2023) to correct spelling mistakes and synonyms. Next, we submitted the list to GBIF to search for occurrence data for each species (rgbif version 3.7.9, Chamberlain et al. 2021, 120923 occurrence points). We excluded records lacking coordinates and submitted the resulting dataset to standard cleaning procedures (CoordinateCleaner version 3.0.1, Zizka et al. 2019) to remove records located in country centroids, the sea, around GBIF headquarters, duplicates or records with equal longitude and latitude, leaving 88932 records across 307 species. Since Taxonstand did not correct all misspelled names and synonyms, we manually corrected names, adding eight species (1158 occurrence points). Further, 21 rare species had been excluded by our strict filtering settings; for these species, we searched occurrences with more relaxed settings, adding 4538 records and 18 species (3 species were not found). We combined these datasets (resulting in 333 species, 94628 occurrence points) and performed a final round of manual cleaning to ensure that all obtained records correspond to the known distribution ranges of the species. Specifically, for each species, we plotted range maps (maptools version 1.1.4, Bivand & Lewin-Koh 2021), compared these maps against the distributions documented in Plants of the World Online (https://powo.science.kew.org/, Figure S1b) and removed records outside of the distribution range of the species (resulting in 90498 records, 333 species, 22 tribes). Next, we subsampled occurrences so that each species was only represented by one occurrence in a 1km grid. This left us with a final dataset of 71122 occurrence records across 22 tribes and 333 species (Table S1), with a median number of 74 occurrence points per species (range 1 – 3037). Across pollination strategies, bee-pollinated species are represented by 63911 records (90%) and shifted species are represented by 7211 records (10%; 4404 records of “generalist”, 2162 records of “nectar-foraging-vertebrate”, 645 records of “food-body-foraging-vertebrate” syndrome).

To investigate the environmental context of pollinator shifts, we chose to focus on elevation, temperature and precipitation since all of these variables can significantly impact the flower

visitation activity and abundance of bee pollinators and may hence be conducive to pollinator shifts (Cruden, 1972, Dellinger et al. 2023). Accordingly, we downloaded layers for mean annual temperature (bio1), mean annual precipitation (bio12, Notes S2) and elevation from CHELSA v2.1 (http://chelsa-climate.org/) at 1 km resolution (Karger et al. 2017). We extracted the respective value for each occurrence record and calculated the median value per species for analyses in a phylogenetic context. The final dataset is available in the online repository phaidra (https://phaidra.univie.ac.at/o:2096899). Note that elevation and mean annual temperature are strongly correlated ($r > -0.8$), we hence only included one at a time in our models (to avoid bias due to autocorrelation, Figure S1a).

<u>Do shifts away from bee pollination associate with montane environments?</u>

To assess whether high-elevation occurrence is linked to non-bee pollination in Melastomataceae, we used boxplots to compare the elevational distribution of bee-pollinated and shifted species, as well as for the four pollination syndromes and elevation. To account for latitudinal effects (i.e., depressed elevation zones at higher latitudes), we plotted pollination systems against elevation and latitude using *ggplot2* (v3.5.1, Wickham 2016). We then applied phylogenetic ANOVAs using the function *phylANOVA* (phytools, version 2.1.1, Revell 2024) to test whether pollination systems differ in elevation. Next, to explore the impact of median elevation, latitude and/or annual precipitation on pollination system, we fitted binary phylogenetic generalized linear mixed models (binaryPGLMM, *binaryPGLMM*, ape version 5.8, Paradis & Schliep 2019). We built an initial binaryPGLMM model including interaction effects among elevation, latitude, and mean annual precipitation. We used stepwise model selection to only retain significant parameters and interaction effects (elevation and latitude in our case) and plotted the expected value of the response variable to evaluate model fit (mu, Figure S2a).

Since median values do not capture the range of environmental conditions that species with wider distribution ranges may encounter, we additionally performed a random sampling approach of the occurrence data to include environmental variables for single occurrence points per species. Specifically, 100 times, we randomly sampled a single occurrence point per species and reran binaryPGLMMs to evaluate the effect of elevation on pollination system. We calculated the percentage of runs where p-values were smaller than 0.05 (indicating significant differences in the elevational distribution among pollination systems, 100% of the runs revealed a significant effect of elevation on pollination system).

We used Whittaker biomes (Whittaker 1975, Ricklefs 2008, plotbiomes, Stefan & Levin 2021) to further explore whether bee-pollinated and shifted species occur in different environments. We used Chi-square statistics to test for differences in biome occupation. To visualize the relative contribution of different biomes to Chi-square statistics, we plotted standardized residuals using correlation plots (corrplot version 0.92, Wei & Simko 2021). Standardized residuals higher than +/- 2 indicate a strong contribution of the respective biome.

Did niche expansion into mountain habitats precede pollinator shifts?

To explore the three different scenarios of evolutionary pollinator and environment shifts (Figure 1), we ran ancestral state reconstructions for both pollination system and elevation (which we determined as most important factor explaining the distribution of pollination systems). We chose to use the four different pollination syndromes ("bee", "nectar-foraging vertebrate", "food-body foraging vertebrate", "generalist") for the initial reconstruction of pollination systems since shifts into each syndrome require different modifications in floral morphology, and reconstructing them all together as "shifted" might artificially inflate shift proportions at internal nodes. To make reconstructions comparable with elevation (a quantitative trait), we chose to categorize elevation into four broad categories: below 500 meters, 500 – 1000 meters, 1000 – 1500 meters, above 1500 meters. This categorization is based off the observation that most pollinator shifts happen above 1000 meters (Figure 2). To ascertain that this categorization retrieved elevation-pollination relationships as reported before, we repeated binaryPGLMMs using pollination system as binary response and categorized elevation as predictor. For ancestral character estimation for pollination system we ran three different MuSSE models (SYM, ARD, Constraint) using the function *make.musse* (diversitree version 0.10.0 FitzJohn 2012) following Kopper et al. 2024. The constraints for transition rates were determined based on our knowledge of the family (Dellinger et al. 2022). For elevation we ran two different MuSSE models (SYM, ARD). We compared model fit using AIC scores (Table S2) and chose the model with the lowest score for either trait (SYM for pollination system and ARD for elevation). For visualization and summary statistics, we binarized pollination system and elevation to have sufficient evolutionary replication and sample sizes per group (binary variable: bee-pollinated/shifted; lowland/montane).

We plotted the reconstructions on the phylogeny for visual interpretation of the sequence of pollinator shifts and elevation shifts. To further explore the sequence of elevation and pollinator shifts (Figure 1), we extracted the reconstructed probability of having shifted pollinators and elevation as well as node ages for all nodes descending from tips with pollinator shifts, until

reaching 100% probability of being bee-pollinated. If only the tip showed 100% probability of having shifted, we used the tip values of this species. If sister species shared a lower node with 100% probability of having shifted, we used the value of the last shared node for this clade with 100% probability of having shifted. We plotted the extracted probabilities for each shifted clade (pollinator shift probability on the x-axis, elevation shift probability on the y-axis) using three different thresholds to define mountain species (500 meters, 1000 meters, 1500 meters, Figure 3b - d), with the expectation that a 1:1 regression line would signify joint shifts in elevation and pollination system (Figure 1). If clade-specific regression lines are above the 1:1-line and the intercept is high, elevation shifts likely preceded pollinator shifts (i.e., higher probability of being montane while still being bee-pollinated, Figure 1a). If clade-specific regression lines are below the 1:1-line and intercepts are negative (with possibly steep slopes only at a high probability of being shifted), pollinator shifts likely happened before elevation shifts (i.e., higher probability of being non-bee pollinated while still being at lower elevations, Figure 1b). In addition, we tested for correlated evolution between pollinator and elevation shifts using the function *fitCorrelationTest* (corHMM version 2.8, Beaulieu et al. 2013) using four syndrome states and four elevational categories.

Since elevation is a naturally continuous trait, we further ran reconstructions treating elevation as a continuous trait using the function *contMap* (phytools, version 2.1.1, Revell 2024) and fitted and *anc.ML* model (phytools, version 2.1.1, Revell 2024) to retrieve the node values. Again, to explore the sequence of elevation and pollinator shifts, we extracted the node values for elevation and pollination system as described above. The intercept gives the elevation where the probability of being shifted increases, positive slopes indicate that clades occur at increasingly higher elevations as shifts occur.

In addition, we used Ornstein-Uhlenbeck-Models (OU-Models) using the function *estimate_shift_configuration* (l1ou, version 1.43 Khabbazian et al. 2016) to test three different hypotheses of elevational evolution in relation to pollination. First, we assigned separate elevational optima for tribes containing species which have shifted pollinators and tribes which remained bee-pollinated. If this model came out as best fitting, it would further support the hypothesis that elevation shifts (whole tribes) precede pollinator shifts (within tribes, Figure 1a). Second, we assigned separate optima for species which shifted pollinators and those which remained bee-pollinated. If this model came out as best fitting, it would support the hypothesis that pollinator shifts are a pre-requisite for elevation shifts (Figure 1b). Third, to explore the elevational relation of different pollination systems, we assigned two separate elevational optima, one for bee-pollinated species and species which shifted to the "generalist" syndrome

and one separate optima for species which either shifted to the "nectar-foraging vertebrate" or to the "food-body-foraging vertebrate" syndrome. We used *estimate_convergent_regimes* (l1ou, version 1.43 Khabbazian et al. 2016) to test for convergent elevational shifts across all models.

Are bee-pollinated species from shifted tribes more likely to occur in environments conducive of pollinator shifts than bee-pollinated species from non-shifted tribes?

Next, to investigate whether bee-pollinated relatives of shifted species are indeed more likely to occur in environments conducive to pollinator shifts (cool and wet mountain habitats, in our case) than bee-pollinated species from non-shifted tribes, we analyzed the environmental associations of bee-pollinated species only (n=280). Using phylogenetic t-tests, we tested whether bee-pollinated species from tribes containing shifts generally occur at higher elevations and cooler and wetter niches than bee-pollinated species from tribes lacking shifts. Additionally, to better capture overall environmental differences, we again used Whittaker biomes (Whittaker 1975, Ricklefs 2008, plotbiomes, Stefan & Levin 2021) to assess whether bee-pollinated species from tribes with pollinator shifts associate with different biomes compared to tribes which remained bee-pollinated as described above.

Are bee-pollinated species in mountains confined to more bee-friendly environments?

To understand whether pollinator shifts may have allowed Melastomataceae to expand their niches into even harsher environments compared to species with the ancestral pollination system, we subset the full dataset to only contain species growing above 1000 meters (the general threshold for pollinator shifts, n = 128). We built a binaryPGLMM model to test whether mean annual temperature and precipitation can determine the probability of being bee-pollinated or shifted, with the expectation that shifted species may occur in even colder and wetter environments than their bee-pollinated relatives (Cruden, 1972, Brito & Sazima, 2012, McCallum et al. 2013, Classen et al. 2015, Cozien et al. 2019, Classen et al., 2020). We used stepwise selection to select the parameters with significant effect on pollination system and plotted the expected value of the response variable, representing the model fit (mu, pollination system, Figure S2b).

Do montane bee-pollinated species have larger flowers and traits facilitating pollen dispersal?

To explore whether montane bee-pollinated species exhibit floral traits potentially increasing attractiveness to pollinators (larger petals) and facilitating pollen release with the scarce

montane bee-pollinators (smooth stamen walls, larger pores), we compared floral traits across bee-pollinated species. Again, we split the dataset into lowland (< 1000 meters) and montane (> 1000 meters) species. We used phylogenetic linear mixed models (*phylolm*, phylolm Ho & Ane 2014) to test for the impact of elevation, mean annual precipitation, latitude and the interaction of latitude and elevation on petal length and pore size, and corresponding binary PGLMMs for the structure of stamens (smooth or ruminate). We used trait states as defined by Kopper et al. (2024). Pore size was determined using the total pore area ($A = \pi \times a \times b$), where 'a' represents half the pore height and 'b' represents half the pore width. We log-transformed petal length and pore size to improve PGLMM model fit and used stepwise model selection to identify significant variables.

Notes S2 Clim Var

BIO1 = Annual Mean Temperature

BIO2 = Mean Diurnal Range (Mean of monthly (max temp - min temp))

BIO3 = Isothermality (BIO2/BIO7) (×100)

BIO4 = Temperature Seasonality (standard deviation ×100)

BIO5 = Max Temperature of Warmest Month

BIO6 = Min Temperature of Coldest Month

BIO7 = Temperature Annual Range (BIO5-BIO6)

BIO8 = Mean Temperature of Wettest Quarter

BIO9 = Mean Temperature of Driest Quarter

BIO10 = Mean Temperature of Warmest Quarter

BIO11 = Mean Temperature of Coldest Quarter

BIO12 = Annual Precipitation

BIO13 = Precipitation of Wettest Month

BIO14 = Precipitation of Driest Month

BIO15 = Precipitation Seasonality (Coefficient of Variation)

BIO16 = Precipitation of Wettest Quarter

BIO17 = Precipitation of Driest Quarter

BIO18 = Precipitation of Warmest Quarter

BIO19 = Precipitation of Coldest Quarter

References

Beaulieu JM, O'Meara BC, Donoghue MJ. 2013. Identifying hidden rate changes in the evolution of a binary morphological character: The corHMM model. *Systematic Biology* **62(5):** 773-784.

Bivand R, Lewin-Koh N. 2023. maptools: Tools for Handling Spatial Objects. http://maptools.r-forge.r-project.org/, https://r-forge.r project.org/projects/maptools/.

Brito VL, Sazima M. 2012. Tibouchina pulchra (Melastomataceae): reproductive biology of a tree species at two sites of an elevational gradient in the Atlantic rainforest in Brazil. *Plant Systematics and Evolution* **298:** 1271-1279.

Chamberlain S, Barve V, Mcglinn D, Oldoni D, Desmet P, Geffert L, Ram K. 2024. rgbif: Interface to the Global Biodiversity Information Facility API. R package version 3.8.1, https://CRAN.R-project.org/package=rgbif.

Classen A, Peters MK, Kindeketa WJ, Appelhans T, Eardley CD, Gikungu MW, Andres H, Thomas N, Steffan-Dewenter I. 2015. Temperature versus resource constraints: which factors determine bee diversity on Mount Kilimanjaro, Tanzania?. *Global Ecology and Biogeography* **24(6):** 642-652.

Classen A, Eardley CD, Hemp A, Peters MK, Peters RS, Ssymank A, Steffan-Dewenter I. 2020. Specialization of plant–pollinator interactions increases with temperature at Mt. Kilimanjaro. *Ecology and evolution* **10(4):** 2182-2195.

Cozien RJ, van der Niet T, Johnson SD, Steenhuisen SL. 2019. Saurian surprise. *Ecology* **100(6):** 1-4.

Cruden RW. 1972. Pollinators in high-elevation ecosystems: relative effectiveness of birds and bees. *Science* **176(4042):** 1439-1440.

Dellinger AS, Kopper C, Kagerl K, Schönenberger J. 2022. Pollination in Melastomataceae: a family-wide update on the little we know and the much that remains to be discovered. In: Goldenberg R, Michelangeli FA, Almeda F, eds. *Systematics, evolution, and ecology of Melastomataceae.* Cham: Springer, 585–607.

Dellinger AS, Hamilton AM, Wessinger CA, Smith SD. 2023. Opposing patterns of altitude-driven pollinator turnover in the tropical and temperate Americas. *The American Naturalist* **202(2):** 152-165.

FitzJohn RG. 2012. Diversitree: Comparative Phylogenetic Analyses of Diversification in R. *Methods in Ecology and Evolution* **3:** 1084-1092, doi:10.1111/j.2041-210X.2012.00234.x

Ho LST & Ane C. 2014. A linear-time algorithm for Gaussian and non-Gaussian trait evolution models. *Systematic Biology* **63(3):** 397-408.

Karger DN, Nobis MP, Normand S, Graham CH, Zimmermann NE. 2023. CHELSA-TraCE21k–high-resolution (1 km) downscaled transient temperature and precipitation data since the Last Glacial Maximum. *Climate of the Past* **19(2):** 439-456.

Khabbazian M, Kriebel R, Rohe K, Ané C. 2016. Fast and accurate detection of evolutionary shifts in Ornstein–Uhlenbeck models. *Methods in Ecology and Evolution* **7(7):** 811-824.

Kopper C, Schönenberger J, Dellinger AS. 2024. High floral disparity without pollinator shifts in buzz-bee-pollinated Melastomataceae. *New Phytologist* **(242)5:** 2322-2337.

McCallum KP, McDougall FO, Seymour RS. 2013. A review of the energetics of pollination biology. *Journal of Comparative Physiology* **183:** 867-876.

Paradis E, Schliep K. 2019. ape 5.0: an environment for modern phylogenetics and evolutionary analyses in R. *Bioinformatics* **35:** 526--528.

Penneys DS, Almeda F, Reginato M, Michelangeli FA, Goldenberg R, Fritsch PW, Stone RD. 2022. A new Melastomataceae classification informed by molecular phylogenetics and morphology. In: Goldenberg R, Michelangeli FA, Almeda F, eds. *Systematics, evolution, and ecology of Melastomataceae.* Cham, Switzerland: Springer, 109 165.

Reginato M, Almeda F, Michelangeli FA, Goldenberg R, Fritsch PW, Stone RD, Penneys DS. 2022. Historical biogeography of the Melastomataceae. In:

Goldenberg R, Michelangeli FA, Almeda F, eds. *Systematics, evolution, and ecology of Melastomataceae.* Cham, Switzerland: Springer, 87–105.

Revell L. 2024. "phytools 2.0: an updated R ecosystem for phylogenetic comparative methods (and other things)." *PeerJ* **12:** e16505. doi:10.7717/peerj.16505.

Ștefan V, Levin S. 2018. plotbiomes: R package for plotting Whittaker biomes with ggplot2 (v1.0.0). Zenodo. https://doi.org/10.5281/zenodo.7145245

Wei T, Simko V. 2024. R package 'corrplot': Visualization of a Correlation Matrix. (Version 0.95), https://github.com/taiyun/corrplot.

Whittaker RH. 1975. Communities and Ecosystems, Macmillan Publishing Co. Inc., New York, New York.

Wickham H. 2016. ggplot2: Elegant Graphics for Data Analysis. Springer-Verlag New York. ISBN 978-3-319-24277-4, https://ggplot2.tidyverse.org.

Zhang J, Qian H. 2023. U.Taxonstand: An R package for standardizing scientific names of plants and animals. *Plant Diversity* **45(1):** 1-5.

Zizka A, Silvestro D, Andermann T, Azevedo J, Duarte Ritter C, Edler D, Farooq H, Herdean A, Ariza M, Scharn R, Svanteson S, Wengstrom N, Zizka V, Antonelli A. 2019. "CoordinateCleaner: standardized cleaning of occurrence records from biological collection databases." Methods in Ecology and Evolution, -7. doi:10.1111/2041-210X.13152, R package version 3.0.1, https://github.com/ropensci/CoordinateCleaner.
