# Supplementary material for: Mountain colonization precedes shifts away from bee pollination in Melastomataceae
Source: New Phytol. 2025 Jun 8;247(3):1474–92. doi: 10.1111/nph.70273 (PMC12222925; doi:10.1111/nph.70273)
Supplement: Supplementary file 3 — Table S2 AIC of MuSSE models. Table S3 BinaryPGLMM full model for pollination syndromes using elevation, latitude and annual precipitation. Table S4 BinaryPGLMM reduced model for pollination system using elevation and latitude. Table S5 pBIC of OU models testing for elevational optima. Table S6 Phylogenetic t‐test using elevation. Table S7 Phylogenetic t‐test for bee‐pollinated species from shifted and nonshifted tribes below 1000 m using annual mean temperature. Table S8 Phylogenetic t‐test for bee‐pollinated species from shifted and nonshifted tribes below 1000 m using annual precipitation. Table S9 Phylogenetic t‐test for bee‐pollinated species from shifted and nonshifted tribes above 1000 m using annual mean temperature. Table S10 Phylogenetic t‐test for bee‐pollinated species from shifted and nonshifted tribes above 1000 m using annual precipitation. Table S11 BinaryPGLMM full model for pollination system above 1000 m using annual mean temperature and annual precipitation. Table S12 BinaryPGLMM reduced model for pollination system above 1000 m using annual mean temperature. Table S13 PGLMM full model for petal length of bee‐pollinated species using elevation, latitude and annual precipitation. Table S14 PGLMM reduced model for petal length of bee‐pollinated species using elevation and latitude. Table S15 PGLMM full model for pore size of bee‐pollinated species using elevation, latitude and annual precipitation. Table S16 PGLMM reduced model for pore size of bee‐pollinated species using elevation and latitude. Table S17 BinaryPGLMM full model for structure of thecal wall using elevation, latitude and annual precipitation. Please note: Wiley is not responsible for the content or functionality of any Supporting Information supplied by the authors. Any queries (other than missing material) should be directed to the New Phytologist Central Office. [file NPH-247-1474-s001.pdf]

***New Phytologist* Supporting Information**

Article title: Mountain colonization precedes shifts away from bee pollination in Melastomataceae
Authors: Constantin Kopper, Jürg Schönenberger, Agnes S. Dellinger
Article acceptance date: 14 May 2025

The following Supporting Information is available for this article:

Table S1 GBIF ccurrence points of the 333 species included in this study. [See separate Excel file]

Table S2 AIC of MuSSE models. Left models for syndromes. Right models for elevation

Model	AICw
ARD	0.00000344
SYM	0.99999656
Constraint	0.00000000

Model	AICw
SYM	0.01
ARD	0.99

Table S3 BinaryPGLMM full model for pollination syndromes using elevation, latitude and annual precipitation.

Fixed effects	Value	Std.Error	Zscore	Pvalue
(Intercept)	-7.5145	2.2711	-3.3087	0.0009
ele1	0.0032	0.0007	4.4169	1.0015e-05
abs(decimalLatitude)	0.1433	0.0722	1.9839	0.0473
wc2.1_30s_bio_12	0.0006	0.0005	1.1449	0.2523
ele1:abs(decimalLatitude)	-3.8882	5.0469-e05	-0.7704	0.4411

Table S4 BinaryPGLMM reduced model for pollination system using elevation and latitude.

Fixed effects	Value	Std.Error	Zscore	Pvalue
(Intercept)	-5.5400	1.4788	-3.7463	0.0002
ele1	0.0027	0.0005	5.6950	1.2338e-08
abs(decimalLatitude)	0.0856	0.0372	2.3020	0.0213

Table S5 pBIC of OU models testing for elevational optima.

	pBIC scores
Shifted Species	5464.15
Shifted Species Conv	5451.59
Shifted NFV FFV	5363.00
Shifted NFV FFV Conv	5355.78
Shifted Tribes	5320.30
Shifted Tribes Conv	5310.58

Table S6 Phylogenetic t-test using elevation.

Parameter	Value
t	20.56
df	277
p	1.1e-57
phylogenetic mean difference	772.71
sig^2	5771.42
Lambda	0
logLik	-2201.56

Table S7 Phylogenetic t-test for bee pollinated species from shifted and non-shifted tribes below 1000 m using annual mean temperature.

Parameter	Value
t	116.424875142502
df	187
p	1.89948908631084e-167
phylogenetic mean difference	23.245143092692
sig^2	0.110555675541005
Lambda	0
logLik	-461.941135473529

Table S8 Phylogenetic t-test for bee pollinated species from shifted and non-shifted tribes below 1000m using annual precipitation.

Parameter	Value
t	47.9923262727943
df	187
p	4.4715638535554e-107
phylogenetic mean difference	2175.14280204462
sig^2	5696.91976094233
Lambda	0
logLik	-1492.68277190982

Table S9 Phylogenetic t-test for bee pollinated species from shifted and non-shifted tribes above 1000 m using annual mean temperature.

Parameter	Value
t	2.22208969648941
df	87
p	1.25973470694494e-37
phylogenetic mean difference	17.7286299040786
sig^2	0.145772199062103
Lambda	0.482212737877057
logLik	-209.210583212937

Table S10 Phylogenetic t-test for bee pollinated species from shifted and non-shifted tribes above 1000 m using annual precipitation.

Parameter	Value
t	26.2407419308008
df	87
p	4.23222715338207e-43
phylogenetic mean difference	1853.52508882604
sig^2	7793.11873607655
Lambda	0.00370367472443903
logLik	-710.706506076161

Table S11 BinaryPGLMM full model for pollination system above 1000 m using annual mean temperature and annual precipitation.

Fixed effects	Value	Std.Error	Zscore	Pvalue
(Intercept)	7.6806	2.4596	3.1227	0.0018
wc2.1_30s_bio_1	-0.5389	0.1344	-4.0100	6.0715-e05
wc2.1_30s_bio_12	0.0002	0.0005	0.4476	0.6544

Table S12 BinaryPGLMM reduced model for pollination system above 1000 m using annual mean temperature.

Fixed effects	Value	Std.Error	Zscore	Pvalue
(Intercept)	7.8311	2.4055	3.2555	0.0011
wc2.1_30s_bio_1	-0.5209	0.1278	-4.0765	4.5722e-05

Table S13 PGLMM full model for petal length of bee-pollinated species using elevation, latitude and annual precipitation.

	Value	Std.Error	Zscore	Pvalue
(Intercept)	1.6074	0.2417	6.6502	2.9266e-11
ele1	0.0005	0.0001	4.2458	2.1779e-05
abs(Latitude)	0.0174	0.0083	2.0884	0.0368
wc2.1_30s_bio_12	7.7916e-05	7.4831e-05	1.0412	0.2978
ele1:abs(Latitude)	-2.2803e-05	8.6914e-06	-2.6236	0.0087

Table S14 PGLMM reduced model for petal length of bee-pollinated species using elevation and latitude.

	Value	Std.Error	Zscore	Pvalue
(Intercept)	1.8137	0.1389	13.0625	5.3943e-39
ele1	0.0004	0.0001	4.1149	3.8735e-05
abs(Latitude)	0.0142	0.0078	1.8347	0.0666
ele1:abs(Latitude)	-2.1710e-05	8.6284e-06	-2.5161	0.0119

Table S15 PGLMM full model for pore size of bee-pollinated species using elevation, latitude and annual precipitation.

	Value	Std.Error	Zscore	Pvalue
(Intercept)	-1.7781	0.3953	-4.4978	6.8650e-06
ele1	0.0004	0.0002	2.4511	0.0142
abs(Latitude)	0.0190	0.0145	1.3081	0.1908
wc2.1_30s_bio_12	3.7932e-06	0.0001	0.0289	0.9769
ele1:abs(Latitude)	-4.6518e-05	1.5227e-05	-3.0550	0.0023

Table S16 PGLMM reduced model for pore size of bee-pollinated species using elevation and latitude.

	Value	Std.Error	Zscore	Pvalue
(Intercept)	-1.7691	0.2118	-8.3516	0,0007
ele1	0.0004	0.0002	2.5085	0.0121
abs(Latitude)	0.0189	0.0135	1.4008	0.1613
ele1:abs(Latitude)	-4.6439e-05	1.4993e-05	-3.0975	0.0020

Table S17 BinaryPGLMM full model for structure of thecal wall using elevation, latitude and annual precipitation.

Fixed effects	Value	Std.Error	Zscore	Pvalue
(Intercept)	0.3191	1.4851	0.2148	0.8299
ele1	0.0007	0.0005	1.2275	0.2196
abs(decimalLatitu	0.0188	0.0427	0.4394	0.6603
wc2.1_30s_bio_12	0.0003	0.0004	0.8840	0.3767
ele1:abs(decimalL	-1.8860e-06	4.5422e-05	-0.0415	0.9669
